# A Comprehensive Total Body Perfusion Strategy vs Conventional Techniques in Elective Aortic Arch Surgery: A Comparative Analysis

**DOI:** 10.1093/icvts/ivag145

**Published:** 2026-05-26

**Authors:** Annesimone Lotfalla, Samuel Heuts, Rick Schreurs, Jos G Maessen, Leon Schurgers, Erik Körver, Sander Verheule, Roberto Lorusso, Michael Jacobs, Elham Bidar

**Affiliations:** Department of Cardiothoracic Surgery, Maastricht University Medical Centre (MUMC+), P. Debyelaan 25, Maastricht 6229 HX, The Netherlands; Cardiovascular Research Institute Maastricht (CARIM), Maastricht University, Minderbroedersberg 4-6, 6211 LK Maastricht, The Netherlands; Department of Cardiothoracic Surgery, Maastricht University Medical Centre (MUMC+), P. Debyelaan 25, Maastricht 6229 HX, The Netherlands; Cardiovascular Research Institute Maastricht (CARIM), Maastricht University, Minderbroedersberg 4-6, 6211 LK Maastricht, The Netherlands; Department of Cardiothoracic Surgery, Maastricht University Medical Centre (MUMC+), P. Debyelaan 25, Maastricht 6229 HX, The Netherlands; Cardiovascular Research Institute Maastricht (CARIM), Maastricht University, Minderbroedersberg 4-6, 6211 LK Maastricht, The Netherlands; Department of Cardiothoracic Surgery, Maastricht University Medical Centre (MUMC+), P. Debyelaan 25, Maastricht 6229 HX, The Netherlands; Cardiovascular Research Institute Maastricht (CARIM), Maastricht University, Minderbroedersberg 4-6, 6211 LK Maastricht, The Netherlands; Cardiovascular Research Institute Maastricht (CARIM), Maastricht University, Minderbroedersberg 4-6, 6211 LK Maastricht, The Netherlands; Department of Cardiothoracic Surgery, Maastricht University Medical Centre (MUMC+), P. Debyelaan 25, Maastricht 6229 HX, The Netherlands; Cardiovascular Research Institute Maastricht (CARIM), Maastricht University, Minderbroedersberg 4-6, 6211 LK Maastricht, The Netherlands; Cardiovascular Research Institute Maastricht (CARIM), Maastricht University, Minderbroedersberg 4-6, 6211 LK Maastricht, The Netherlands; Department of Cardiothoracic Surgery, Maastricht University Medical Centre (MUMC+), P. Debyelaan 25, Maastricht 6229 HX, The Netherlands; Cardiovascular Research Institute Maastricht (CARIM), Maastricht University, Minderbroedersberg 4-6, 6211 LK Maastricht, The Netherlands; Department of Vascular Surgery, Maastricht University Medical Centre (MUMC+), P. Debyelaan 25, 6629 HX Maastricht, The Netherlands; Department of Cardiothoracic Surgery, Maastricht University Medical Centre (MUMC+), P. Debyelaan 25, Maastricht 6229 HX, The Netherlands; Cardiovascular Research Institute Maastricht (CARIM), Maastricht University, Minderbroedersberg 4-6, 6211 LK Maastricht, The Netherlands

**Keywords:** aortic arch surgery, myocardial protection, organ protection, beating heart

## Abstract

**Objectives:**

Aortic arch surgery traditionally relies on hypothermic circulatory and cardioplegic arrest, exposing myocardium and visceral organs to ischaemia-reperfusion injury. We developed a total body perfusion (TBP) strategy providing continuous cerebral, myocardial, and visceral perfusion during arch reconstruction. This study compares outcomes between conventionally and TBP-treated patients.

**Methods:**

Analysis included 121 consecutive elective aortic arch replacements (2014-2025). From June 2022, patients (*n* = 38) were intended for TBP; earlier cases (*n* = 83) underwent conventional techniques. TBP comprised continuous warm myocardial, selective cerebral, and retrograde visceral perfusion. Outcomes included intraoperative characteristics, biochemical markers, and clinical outcomes.

**Results:**

Baseline demographics were similar (male 65.8% vs 53.0%, *P* = .188; median age 63.9 [IQR 60.5-72.4] vs 66.1 [IQR 58.7-70.6] years, *P* = .915). TBP-patients presented with greater surgical complexity (more concomitant procedures: 65.8% vs 45.8%, *P* = .041; chronic dissection: 18.4% vs 0.0%, *P* < .001; active infection at time of surgery: 15.8% vs 3.6%, *P* = .027). TBP reduced myocardial ischaemia time (median 65.0 [IQR 39.0-98.5] vs 122.0 [IQR 93.0-170.0] min, *P* < .001) and circulatory arrest use (18.4% vs 86.7%, *P* < .001), and demonstrated lower peak lactate levels (median 2.2 [IQR 1.5-4.6] vs 3.9 [IQR 2.9-5.2] mmol/L, *P* < .001), shorter ICU stay (median 2.0 [IQR 1.0-7.0] vs 4.0 [IQR 3.0-8.0] days, *P* = .006), and improved one-year survival (92.1% vs 72.3%, *P* = .025). Multivariable analysis confirmed TBP’s independent survival benefit (HR = 0.187, 95% CI 0.044-0.807, *P* = .025). Sensitivity analyses were directionally consistent.

**Conclusions:**

TBP appeared as a feasible and safe approach in elective aortic arch surgery, associated with improved clinical outcomes and offering comprehensive organ protection. TBP may expand surgical candidacy in high-risk patients.

## Graphical Abstract

**Figure:**
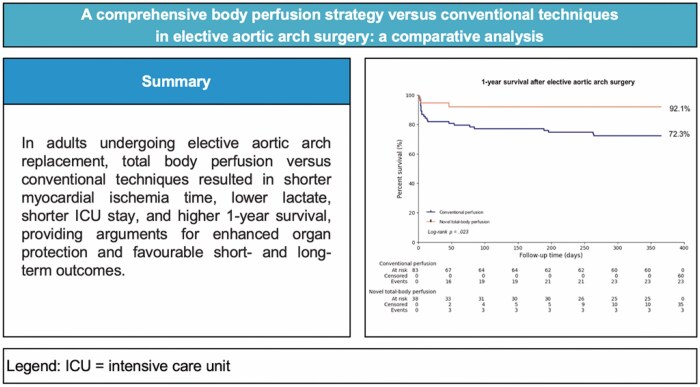

## INTRODUCTION

Aortic arch surgery represents one of the most technically and physiologically challenging procedures within the field of cardiovascular surgery, necessitating a delicate equilibrium between adequate surgical exposure and the imperative to mitigate ischemic injury to vital organs.^1^ Achieving optimal exposure of the aortic arch requires a bloodless surgical field that is accomplished through temporary interruption of systemic perfusion, thereby placing the brain, heart, and visceral organs at considerable risk of ischemic and reperfusion injury.^2^ Historically, considerable attention has been directed toward improving cerebral protection, culminating in the widespread adoption of selective antegrade cerebral perfusion (SACP) under hypothermic circulatory arrest as the standard of care.^3^^,^^4^ This approach has markedly reduced the incidence of neurological complications.^3^^,^^5^^,^^6^ In parallel, although visceral organs generally tolerate ischemia better than neurons, efforts to protect visceral organs have gained momentum, particularly through the increasing use of lower body perfusion techniques at moderate hypothermia, which help to mitigate the risks of renal and bowel ischemia during prolonged or complex procedures.^7–9^

The heart, subjected to cardioplegic arrest during surgery, remains vulnerable to ischemia/reperfusion injury that can result in significant myocardial damage, arrhythmias, and low cardiac output syndrome.^10–12^ This risk is particularly pronounced in patients with pre-existing reduced left ventricular function, who are more vulnerable to a further decrease in myocardial function and subsequent poor postoperative outcomes.^13–15^ Furthermore, in cases involving prolonged surgeries, such as when concomitant cardiac procedures are required, the risk of myocardial ischemic burden is further amplified.^15–17^ Although myocardial protection strategies exist to limit ischemic time, the challenge of achieving comprehensive, total body protection during aortic arch reconstruction remains unmet.^18^

At our centre, we developed and implemented a comprehensive strategy designed to provide continuous whole-body perfusion.^19^ By maintaining uninterrupted blood flow to the cerebral, myocardial and systemic circulations, this technique seeks to minimize physiological derangement and improve outcomes. The present study undertakes a comparative analysis between patients treated with a total body perfusion (TBP) approach and a historical cohort receiving conventional perfusion techniques during aortic arch surgery.

## METHODS

### Study design and patient population

This observational cohort study was conducted at Maastricht University Medical Centre (MUMC+), a Dutch tertiary academic centre serving as a regional referral centre for aortic surgeries. All consecutive adult patients undergoing elective hemiarch or total arch replacement between January 2014 and April 2025 were included. A comprehensive perfusion strategy was introduced in June 2022 and used routinely thereafter. Patients undergoing emergency surgery for acute type A aortic dissection and those receiving isolated ascending aortic replacement without arch involvement were excluded.

The study was approved by the medical ethics committee of Maastricht (registration number METC-2019-1235, October 2019), with informed consent waived due to its retrospective, observational nature.

### Surgical technique and perfusion strategy

Before June 2022, aortic arch replacement was performed using conventional techniques involving cardiac and circulatory arrest under hypothermia with SACP, reflecting standard practice, but influenced by surgeon preferences.

**Figure 1.**
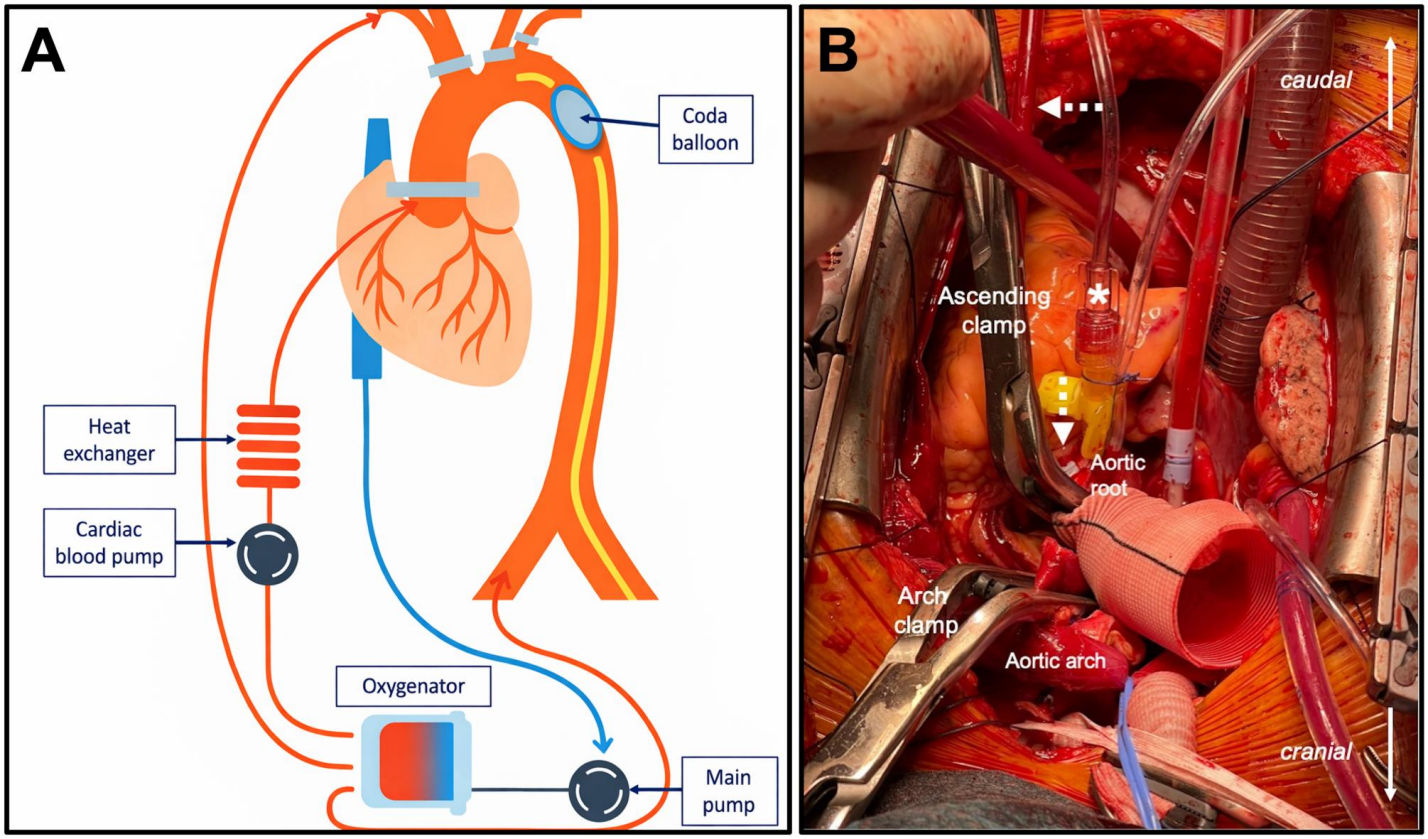
(A) Schematic overview of the TBP strategy: cerebral perfusion via the right axillary artery, warm antegrade myocardial perfusion through a heat exchanger connected to a separate pump, and retrograde visceral perfusion via the femoral artery using an intra-aortic occlusion device. (B) Intraoperative view demonstrating aortic root pressure monitoring needle (asterisk) for continuous measurement of mean aortic root pressure during warm antegrade myocardial perfusion (perfusion cannula indicated by interrupted arrow), enabling flow titration.

From June 2022 onward, elective procedures employed a previously described technique involving continuous warm myocardial perfusion,^19^ performed by a dedicated team of surgeons and perfusionists, supporting procedural consistency, training, and reflecting the progression of a focused learning curve. Although each patient presented with individual anatomical and procedural variations, the following describes the general strategy employed (**Figure 1A**).

All procedures included neurophysiological monitoring, including transcranial Doppler (TCD) and near-infrared spectroscopy. Cardiopulmonary bypass (CPB) was established via separate axillary and femoral arterial cannulation; additional left axillary access was used for FET-cases to facilitate an extra-anatomic anastomosis with the ascending aortic prosthesis. Femoral access was achieved using a single-site EndoReturn cannula, allowing retrograde perfusion and transoesophageal echocardiography (TEE-)guided Coda-balloon placement in the descending aorta to isolate the distal circulation and minimize embolic risk. Venous drainage was typically achieved through standard 2-stage right atrial cannulation.

After inducing mild hypothermia, early debranching of supra-aortic vessels was performed with SACP of right axillary artery and left common carotid arteries, titrated using TCD-monitoring.

Cardioplegic arrest was initiated in cases requiring concomitant cardiac procedures (eg, aortic valve or root surgery). Following de-airing of cardiac chambers, continuous antegrade myocardial perfusion was established using the Medtronic MYOtherm XP system connected to a dedicated circuit and heat exchanger. Perfusion was delivered via an aortic root needle after clamping the aortic graft or native aorta distal to the needle insertion site. Warm blood, differing by a maximum of 6 degrees from systemic circulation temperature, was used for myocardial perfusion. Mean aortic root pressure was continuously monitored via a separate needle inserted into the aortic root (**Figure 1B**), enabling real-time intraluminal pressure monitoring throughout myocardial perfusion. Myocardial perfusion flow was maintained at 300-500 mL/min and titrated to root pressure, electrocardiographic (ECG) findings and TEE-based assessment of left ventricular (LV) size and function. Target mean root pressure was maintained between 70 and 90 mm Hg, adjusted for the presence and severity of aortic valve insufficiency. In case of LV distention or persistent signs of myocardial ischemia despite flow adjustment, cardioplegia was used.

Lower body perfusion was initiated by inflating the Coda-balloon positioned in the descending thoracic aorta. A low-pressure, lactate-guided retrograde flow was maintained, generally at 2 L/min.

This configuration provided uninterrupted cerebral, myocardial, and visceral perfusion during arch reconstruction. Rewarming commenced after distal anastomosis completion, followed by reintegration of cardiac and systemic circulations. Final procedural steps included completion of the proximal and supra-aortic anastomoses and subsequent weaning from CPB.

### Outcomes and definitions

The study was designed as an era-based comparison reflecting a strategy-level change in institutional perfusion practice. Patients treated from June 2022 onward (TBP-group, the intervention) were compared with those treated before (conventional group, controls), marking the introduction of the new perfusion strategy. Outcomes included intraoperative parameters, biochemical markers indicative of myocardial and visceral damage, and clinical outcomes (definitions provided in **Supplementary Material S1**).

Surgical risk was quantified by the European system for operative risk evaluation (EuroSCORE II). Active cardiovascular infection was defined as the presence of mycotic aneurysms, infective endocarditis, and/or prosthetic aortic graft infections at the time of surgical intervention. Chronic aortic dissection was characterized as cases requiring aortic arch reconstruction at least 2 weeks following the initial dissection event.

### Statistical analysis

Continuous variables were expressed as mean ± standard deviation or median [interquartile range] and compared using the Student’s t-test or Mann-Whitney U test as appropriate. Categorical variables were analysed using Chi-square or Fisher’s exact test. Kaplan-Meier and log-rank tests assessed survival. Multivariable Cox regression models identified independent predictors of survival. Model variables were selected based on clinical relevance and statistical significance in univariable analysis, and included age, gender, EuroSCORE II, active cardiovascular infection at time of surgery, and application of the TBP-strategy. Variance inflation factors (VIFs) were calculated and showed no problematic multicollinearity.

Analyses followed a pragmatic, strategy-based intention-to-treat approach, comparing patients according to the intended perfusion strategy at the time of surgical planning, based on institutional practice before and after June 2022. This approach was chosen to reflect real-world strategy implementation and avoid post hoc reclassification based on intraoperative feasibility. Complementary as-treated and per-protocol sensitivity analyses were performed.

Additional sensitivity analyses included restriction to patients treated between 2020 and 2025 to mitigate potential temporal confounding, and to patients undergoing total arch replacement given the greater procedural complexity and expected higher risk profile of this subgroup. Additionally, a landmark analysis at 30 days evaluated early (0-30 days) and late (>30 days) events.

To account for differences in follow-up between groups, follow-up was truncated at 3.5 years post-surgery with censoring thereafter.

Post hoc power for 1-year survival was estimated at 85.4%. Statistical significance was set at *P* < .05. Analyses used SPSS v29. Figures were created using GraphPad Prism v9.

## RESULTS

### Patient characteristics

A total of 121 patients underwent elective aortic arch replacement, with 38 (31.4%) treated after June 2022, when TBP was introduced as the preferred strategy. Of these, 29 (76.3%) effectively received TBP, with deviations due to learning curve, technical/anatomical limitations mainly related to myocardial distention due to aortic valve insufficiency, and team training-dependent logistical constraints (**Table S1**).

Baseline demographics, cardiac function as quantified by left ventricular ejection fraction (LVEF), and surgical risk based on the EuroSCORE II, were similar between groups (**Table 1**). However, TBP-treated patients more often had concomitant cardiac procedures (65.8% vs 45.8%, *P* = .041), chronic aortic dissection (18.4% vs 0.0%, *P* < .001), active cardiovascular infection at the time of surgery (15.8% vs 3.6%, *P* = .027), and total arch replacement (78.9% vs 59.0%, *P* = .033).

**Table 1.** Baseline Characteristics

Parameter	Conventional (*n* = 83)	TBP (*n* = 38)	*P*-value
Demographics			
Male (*n*, %)	44 (53.0%)	25 (65.8%)	.19
Age (years)	66.1 [58.7-70.6]	63.9 [60.5-72.4]	.92
BMI (kg/m^2^)	25.8 [23.2-29.0]	25.8 [23.6-29.8]	.79
EuroSCORE II	5.14 [2.92-9.65]	4.88 [3.13-6.60]	.65
Comorbidities and cardiac function			
Diabetes (*n*, %)	3 (3.6%)	3 (7.9%)	.38
Preoperative serum creatinine (umol/L)	85 [72-106]	87 [69-101]	.71
Preoperative eGFR (mean ± SD, ml/min)	76.4 ± 18.9	79.8 ± 19.7	.36
LVEF (%)	55 [54-58]	55 [47-59]	.15
Operative characteristics			
Reoperation (*n*, %)	19 (22.9%)	8 (21.1%)	.82
Chronic aortic arch dissection (*n*, %)	0 (0.0%)	7 (18.4%)	<.001*
Active infective endocarditis, prosthetic infection, or mycotic aneurysm at time of surgery (*n*, %)	3 (3.6%)	6 (15.8%)	.027*

All values are presented as median [IQR], unless stated otherwise. Values in bold marked with an asterisk (*) denote statistically significant differences between groups (p < .05).

Abbreviations: BMI, Body Mass Index; eGFR, estimated glomerular filtration rate (as estimated by CKD-EPI); IQR, interquartile range; LVEF, left ventricular ejection fraction; SD, standard deviation.

### Intraoperative characteristics

Intraoperative data are summarized in **Table 2**. Total CPB-time did not differ significantly between groups (median 230.0 [IQR 196.5-268.0] vs 228.0 [IQR 194.0-283.0] min, *P* = .800), while myocardial ischemia time was significantly shorter in patients treated with continuous myocardial perfusion (median 65.0 [IQR 39.0-98.5] vs 122.0 [IQR 93.0-170.0] min, *P* < .001). Following the introduction of the TBP-strategy, the use of circulatory arrest decreased significantly (*n* = 7 [18.4%] vs *n* = 72 [86.7%], *P* < .001), with use of higher target temperatures (median 29.8°C [28.0-31.8] vs 25.0°C [24.5-28.0], *P* < .001). These intraoperative differences showed consistent trends across all sensitivity analyses, including per-protocol and as-treated analyses, the 2020-2025 restricted cohort, and analyses limited to total arch replacement (**Tables S2-S5**).

**Table 2.** Intra-Operative Characteristics

Parameter	Conventional(*n* = 83)	TBP (*n* = 38)	*P*-value
Concomitant procedures			
Concomitant aortic root procedure (*n*, %)	30 (36.1%)	15 (39.5%)	.48
Any concomitant cardiac surgery (*n*, %)	38 (45.8%)	25 (65.8%)	.041*
Total arch replacement (*n*, %)	49 (59.0%)	30 (78.9%)	.033*
FET/ET (*n*, %)	31 (38.6%)	14 (39.5%)	.92
Operative parameters			
Lowest temperature (°C)	25.0 [24.5-28.0]	29.8 [28.0-31.8]	<.001*
ECC duration (min)	228.0 [194.0-283.0]	230.0 [196.5-268.0]	.80
Myocardial ischaemia time (min)	122.0 [93.0-170.0]	65.0 [39.0-98.5]	<.001*
Circulatory arrest (*n*, %)	72 (86.7%)	7 (18.4%)	<.001*
Circulatory arrest time (min)	44.0 [26.0-55.0]	0.0 [0.0-15.0]	<.001*

All values are presented as median [IQR], unless stated otherwise. Values in bold marked with an asterisk (*) denote statistically significant differences between groups (p < 0.05).

Abbreviations: ECC, extracorporeal circulation; ET, elephant trunk; FET, frozen elephant trunk; IQR, interquartile range.

### Biochemical outcomes

Intra- and postoperative laboratory values are summarized in **Table 3**. Both peak intra- and postoperative lactate levels were significantly lower in TBP-patients (median 2.2 [IQR 1.5-4.6] vs 3.9 [2.9-5.2] mmol/L, *P* < .001 and median 2.7 [1.5-4.0] vs 3.6 [2.3-6.4] mmol/L, *P* = .017, respectively). Directionally similar reductions in lactate levels were observed across all performed sensitivity analyses, although statistical significance was not uniformly retained in smaller subgroups (**Tables S2-S5**). Markers of cardiac damage or renal dysfunction did not significantly differ between groups.

**Table 3.** Biochemical Outcomes

Parameter	Reference	Conventional (*n* = 83)	TBP (*n* = 38)	*P*-value
Peak lactate levels				
Intraoperatively (mmol/L)	0.6-2.4	3.9 [2.9-5.2]	2.2 [1.5-4.6]	<.001*
48 h postoperatively (mmol/L)		3.6 [2.3-6.4]	2.7 [1.5-4.0]	.017*
48 h postoperatively (including intraoperative values) (mmol/L)		4.8 [3.3-6.9]	2.9 [1.8-5.3]	.002*
Peak concentrations cardiac biomarkers 48 h postoperatively				
CK (U/L)	<160	810.0 [485-1737.0]	800.0 [401.8-1217.8]	.46
CKMB (ug/L)	<2.9	41.1 [27.7-160.7]	38.8 [26.5-76.7]	.26
hsTnT (ng/L)	<14	2573.0 [410.0-11258.0]	1741.5 [817.3-2785.0]	.48
Renal biomarkers				
Peak serum creatinine concentration 48 h postoperatively (umol/L)	50-100	111.0 [86.0-174.0]	103.0 [85.0-129.0]	.24
Maximum creatinine elevation from baseline		22.0 [3.0-67.5]	16.0 [-3.8-44.5]	.137*

All values are presented as median [IQR], unless stated otherwise. Values in bold marked with an asterisk (*) denote statistically significant differences between groups (p < 0.05).

Abbreviations: CK, creatinine kinase; CK-MB, creatinine kinase-M; hsTnT, high-sensitivity troponin T; IQR, interquartile range.

### Clinical postoperative outcomes

**Figure 2.**
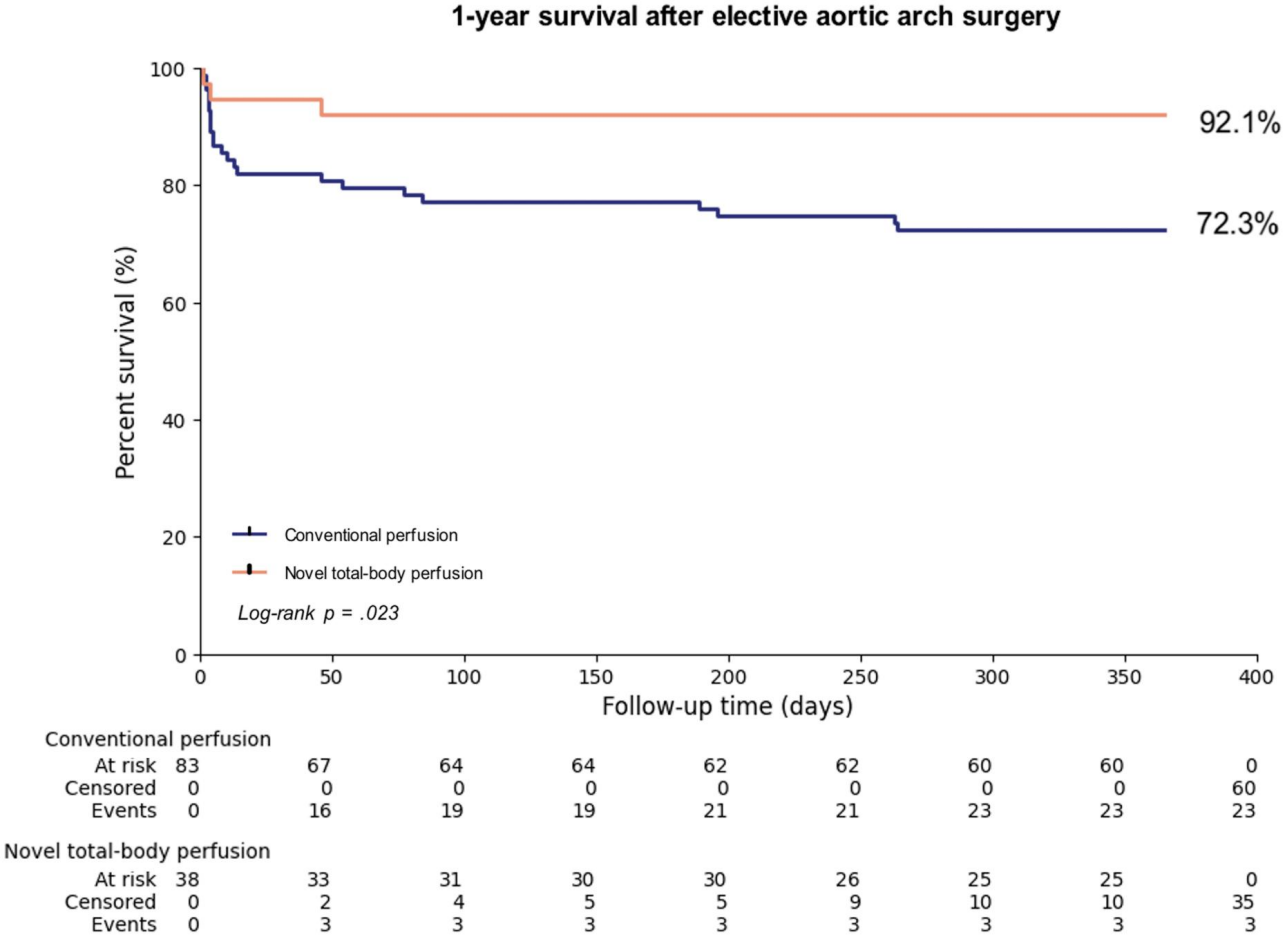
Kaplan-Meier Curve for Survival after 1 Year, Stratified by Perfusion Strategy

In-hospital mortality was lower in the TBP-group (7.9% vs 21.7%, *P* = .063). Thirty-day survival was 94.7% with TBP vs 81.9% with conventional techniques (log-rank *P* = .083). One-year survival was significantly improved in the TBP-group (92.1% vs 72.3%, log-rank *P* = .025), as illustrated by the Kaplan-Meier curve (**Figure 2**). Multivariable Cox-regression analysis adjusting for age, sex, EuroSCORE II, and active infection at time of surgery confirmed a significant association between TBP and improved survival (HR = 0.187, 95% CI 0.044-0.807, *P* = .025) (**Table 4**). Landmark analysis showed that during the first 30 days, survival tended to be higher with TBP (log-rank *P* = .068), whereas among 30-day survivors, no significant difference was observed (log-rank *P* = .178) (**Figure S3**). Across sensitivity analyses the direction of the association between TBP and survival remained consistent, although statistical significance was not uniformly preserved due to reduced sample size and event rates (**Tables S2-S5**, **Figures S1 and S2**).

Additional clinical outcomes are presented in **Table 5**. Incidence of cardiac, renal, respiratory, and gastrointestinal complications did not differ significantly between groups. Permanent neurological deficit occurred less frequently in TBP-treated patients, without statistical significance (2.6% vs 13.3%, *P* = .070). Across all analyses, ICU-stay was longer in conventionally treated patients (4.0 [3.0-8.0] vs 2.0 [1.0-7.0] days, *P* = .006), while total hospital length of stay was comparable (median 10.0 [6.0-17.0] vs 8.0 [6.0-15.3] days, *P* = .455).

**Table 4.** Cox-Regression Analysis for Survival after 1 Year

Variable	Univariable		Multivariable	
	HR (95% CI)	*P*-value	HR (95% CI)	*P*-value
Age	0.987 (0.958-1.018)	.41	0.975 (0.943-1.007)	.13
Female	1.339 (0.620-2.888)	.46	1.475 (0.632-3.444)	.37
EuroSCORE II	1.063 (0.990-1.141)	.093	1.055 (0.876-1.141)	.18
Cardiac infection at time of surgery	1.131 (0.267-4.794)	.87	1.608 (0.324-7.985)	.56
TBP-approach	0.277 (0.083-0.923)	.037*	0.187 (0.044-0.807)	.025*

Values in bold marked with an asterisk (*) denote statistically significant differences between groups (p < 0.05). Abbreviations: CI, confidence interval; HR, hazard ratio; TBP, total body perfusion.

**Table 5.** Clinical Outcomes

Parameter^a^	Conventional (*n* = 83)	TBP (*n* = 38)	*P*-value
Mortality			
In-hospital mortality (*n*, %)	18 (21.7%)	3 (7.9%)	.063
Cardiovascular complications			
Myocardial infarction (*n*, %)	2 (2.4%)	1 (2.6%)	1.000
Respiratory complications			
Pulmonary infection (*n*, %)	13 (15.7%)	8 (21.1%)	.45
Respiratory insufficiency (*n*, %)	12 (14.5%)	3 (7.9%)	.39
Neurological complications			
Permanent neurological dysfunction (*n*, %)	11 (13.3%)	1 (2.6%)	.070
Transient neurological dysfunction (*n*, %)	5 (6.0%)	3 (7.9%)	.71
Total stroke incidence (*n*, %)	16 (19.3%)	4 (10.5%)	.23
Renal and gastrointestinal complications			
Acute renal failure (*n*, %)	19 (22.9%)	5 (13.2%)	.21
Gastrointestinal complications (*n*, %)	8 (9.6%)	1 (2.6%)	.27
ICU management and surgical complications			
ECMO (*n*, %)	7 (8.4%)	2 (5.3%)	.72
ICU readmission (*n*, %)	14 (16.9%)	4 (10.5%)	.36
Rethoracotomy (*n*, %)	15 (18.1%)	4 (10.5%)	.29
Cardiac tamponade (*n*, %)	4 (4.8%)	3 (7.9%)	.68
Length of stay			
ICU length of stay (days)	4.0 [3.0-8.0]	2.0 [1.0-7.0]	.006*
Hospital length of stay (days)	10.0 [6.0-17.0]	8.0 [6.0-15.3]	.46

All values are presented as median [IQR], unless stated otherwise. Values in bold marked with an asterisk (*) denote statistically significant differences between groups (p < 0.05).

Abbreviations: ECMO, extracorporeal membrane oxygenation; ICU, intensive care unit; IQR, interquartile range.

a Detailed definitions of outcomes are provided in the **Supplementary Material (S1)**.

No significant group differences were observed in incidences of postoperative tamponade requiring re-thoracotomy or the need for extracorporeal membrane oxygenation (ECMO) support.

## DISCUSSION

Systemic cooling combined with total circulatory and cardiac arrest remains the gold standard surgical strategy for aortic arch repair. However, advances in perfusion techniques have made selective organ perfusion increasingly feasible. While SACP during circulatory arrest is widely accepted, selective perfusion of the heart and visceral organs has not yet been adopted as a routine approach.

This report describes a comprehensive technique incorporating selective organ perfusion during arch repair, providing signals of clinical benefit. Our findings demonstrate that TBP is a safe and potentially impactful approach to aortic arch surgery.

TBP demonstrated a significant one-year survival improvement (92.1% vs 72.3%, *P* = .025), even after adjusting for key confounders (HR = 0.187, 95% CI 0.044-0.807, *P* = .025). Restricting analyses to the 2020-2025 cohort—thereby minimizing temporal bias from evolving techniques—yielded similar statistically significant findings, suggesting the observed effect is unlikely to be explained solely by temporal improvements and may reflect a consistent effect of the perfusion strategy itself, albeit hypothesis-generating.

Interestingly, these signals of benefit occurred despite the TBP-group presenting with more chronic aortic dissections, active cardiovascular infections, concomitant procedures and total arch replacements—factors typically associated with worse outcomes,^20^^,^^21^ suggesting that TBP might be safely applied in high-risk cases where conventional approaches carry considerable risk. Rather than implying superiority, these findings support the feasibility of applying TBP in demanding clinical scenarios without apparent compromise in safety. A plausible mechanistic explanation may relate to the hypothesis that TBP might extend beyond basic perfusion adequacy by maintaining more physiological conditions during complex aortic arch reconstruction. Such conditions may contribute to a reduced ischemic load and improved and durable organ perfusion, positively affecting clinical outcomes. By avoiding deep hypothermic circulatory arrest in the majority of cases (18.4% vs 86.7%, *P* < .001), as well as cardioplegic arrest, we speculate that TBP may attenuate systemic inflammatory responses and organ dysfunction typically associated with hypothermia and ischemia-reperfusion injury.^11^^,^^12^^,^^22^^,^^23^ Consistent with this hypothesis, the landmark analysis suggested a predominantly early survival benefit, possibly explained by modulation of systemic inflammation and vasoplegia.

Supporting this, TBP-patients had significantly lower intra- and postoperative lactate levels across all sensitivity analyses, suggesting improved maintenance of cellular metabolism and tissue perfusion during the procedure.^24^^,^^25^ This finding is particularly relevant as elevated lactate levels have been associated with poor outcomes in cardiac surgery.^26–28^

Although this study was underpowered for neurological outcomes, the numerically lower stroke rate (2.6% in TBP vs 13.3% in conventional group, *P* = .070) may result from SACP and avoidance of deep hypothermia, both of which are important determinants of neurological complications in arch surgery.^24^^,^^25^ Furthermore, these findings compare favourably to literature reporting stroke rates of 3.5% to 20% depending on technique and case complexity.^23^^,^^25^^,^^29^

Additionally, the study identified technical advantages of TBP. Avoidance of cardioplegic arrest and a significant reduction in myocardial ischemia time may suggest improved myocardial protection. However, absence of significant differences in postoperative myocardial biomarker release renders this benefit less conclusive and underscores necessity for additional investigation. Interestingly, despite more concomitant procedures in the TBP-group, overall CPB times were similar (median 230.0 [IQR 196.5-268.0] vs 228.0 [IQR 194.0-283.0] min, *P* = .800).

In terms of healthcare resource utilization, TBP was associated with significantly shorter ICU-stay (median 2.0 [1.0-7.0] vs 4.0 [IQR 3.0-8.0] days, *P* = .006), a finding consistent across all sensitivity analyses, suggesting a more stable postoperative recovery with potentially fewer complications requiring intensive monitoring. Although total hospital length of stay was similar, reduced ICU-demand has meaningful implications for cost-effectiveness and hospital throughput.

### Limitations

Despite encouraging results, several limitations warrant mention. First, the study design is observational and era-based, introducing potential temporal bias related to evolving techniques and learning curves. To address this, sensitivity analyses restricting the control group to more recent years were performed to evaluate temporal impact on key outcomes, yielding consistent trends and suggesting the findings are not solely driven by chronological differences. Nevertheless, residual bias cannot be completely eliminated.

Second, the non-randomized design limits causal inference, and unmeasured confounders may exist. Additionally, small sample sizes may limit the generalizability to larger populations and reduce the statistical power of some analyses, particularly for rarer outcomes. Differences in follow-up duration and censoring patterns between eras may further influence hazard ratio estimation in Cox regression models despite follow-up truncation and sensitivity analyses. Accordingly, results should be interpreted as associative rather than confirmatory, and findings are best considered hypothesis-generating.

Third, although analyses followed an intention-to-treat analytical approach reflecting real-world implementation of a strategy-level change, it is noteworthy that in 23.7% of the cases the intended intervention was not administered—mainly due to logistical or technical constraints like myocardial distention or venting difficulties. To address this, complementary as-treated and per-protocol analyses were performed and yielded directionally consistent results, while highlighting the need for further evaluation of implementation barriers. Future studies should not only aim to replicate the intervention but also systematically investigate the operational and technical challenges encountered during implementation.

Fourth, the high mortality rate in the conventional cohort likely reflects a more complex case mix, including high prevalence of concomitant procedures, root involvement, and reoperations.^30^^,^^31^ However, TBP-mortality rates were within or below those reported in comparable studies,^31–33^ suggesting at least comparable, if not improved, safety. Interpretation of results should balance these observations with awareness of the underlying case mix and institutional characteristics.

Finally, subgroup and sensitivity analyses were necessarily limited by sample size and event rates. Loss of statistical significance should, therefore, be interpreted in the context of reduced power and not necessarily as absence of effect.

### Future directions

TBP may offer a path toward more comprehensive organ protection and improved surgical outcomes in aortic arch repair. Our findings align with an evolving paradigm in arch surgery, where the focus is shifting beyond cerebral protection toward comprehensive whole-organ preservation. While cerebral and lower-body perfusion are increasingly adopted, myocardial protection remains inconsistently addressed. Current European guidelines provide a moderate (IIb) recommendation against cardioplegic arrest^33^ highlighting the relevance of our findings as a potential foundation for such a recommendation. Future validation in larger, multicentre populations and randomized trials is needed to reduce confounding and further define TBP’s role across subgroups. In parallel, preclinical studies could elucidate the mechanistic underpinnings of TBP’s hypothesized protective effects, including the roles of inflammation, ischemia-reperfusion injury, and cardiac, visceral and neurological protection. Successful implementation of TBP will require standardized protocols and dedicated multidisciplinary teams comprising surgeons, anesthesiologists, perfusionists and clinical neurophysiologists. Ultimately, the development of personalized perfusion strategies, tailored to individual patient characteristics, constitutes an essential next step for advancing this field.

## CONCLUSION

This study demonstrates feasibility and safety of the TBP strategy in elective aortic arch surgery and suggests it represents a promising perfusion strategy evolution. TBP implementation was associated with signals of improved survival, lower lactate levels, and decreased ICU utilization, with benefits pronounced in high-risk and complex cases, indicating that TBP may expand the therapeutic window for patients previously considered borderline candidates for surgery. These findings should be interpreted as hypothesis-generating and support further prospective evaluation of TBP as a strategy for comprehensive organ protection.

## Supplementary Material

ivag145_Supplementary_Data

## Data Availability

The data underlying this article will be shared on reasonable request to the corresponding author.
