# Supplementary material for: A Comprehensive Total Body Perfusion Strategy vs Conventional Techniques in Elective Aortic Arch Surgery: A Comparative Analysis
Source: Interdiscip Cardiovasc Thorac Surg. 2026 May 26;41(6):ivag145. doi: 10.1093/icvts/ivag145 (PMC13310033; doi:10.1093/icvts/ivag145)
Supplement: ivag145_Supplementary_Data [file ivag145_supplementary_data.docx]

**Supplementary material S1: clinical outcome definitions**

Cardiovascular complications

*Myocardial infarction*

Defined according to The Society of Thoracic Surgeons (STS) criteria: troponin level >10 times the upper limit of normal (99th percentile) when baseline troponin was normal, AND at least one of the following:

- New pathological Q-waves or left bundle branch block on ECG
- Angiographic evidence of new graft or native coronary artery occlusion
- Imaging evidence of new loss of viable myocardial tissue or new regional wall motion abnormalities

*Cardiac tamponade*

As confirmed by echocardiographic assessment demonstrating hemodynamically significant pericardial effusion and/or diagnosed during surgical re-exploration

Neurological complications

*Permanent neurological deficit (PND)*

Neurologist-confirmed postoperative stroke occurring during hospital admission for the current intervention (excluding TIA). Stroke is defined as permanent neurological dysfunction resulting from focal ischemia of the brain, spinal cord, or retina, caused by acute infarction of neurological tissue due to thrombosis, embolism, systemic hypoperfusion, or hemorrhage.

*Transient neurological deficit (TND)*

Neurologist-confirmed central neurological deficit (stroke) occurring during the postoperative period, but without residual deficits at discharge. This includes Transient Ischemic Attack (TIA).

Pulmonary complications

*Pulmonary infection*

Defined as positive respiratory tract cultures with pathogenic organisms and/or initiation of targeted antimicrobial therapy for suspected or confirmed pneumonia.

*Respiratory insufficiency*

Requirement for reintubation and mechanical ventilation following initial successful extubation.

Renal complications

*Acute renal failure*

Requirement for renal replacement therapy in patients without preoperative renal dysfunction and/or peak serum creatinine concentration >177 µmol/L combined with doubling of preoperative creatinine baseline values.

Gastrointestinal complications

- Gastrointestinal hemorrhage requiring blood product transfusion, endoscopic intervention, or surgical management
- Mesenteric ischemia
- Acalculous cholecystitis

**Supplementary Table S1: reasons for non-implementation of TBP approach**

|  | Number of patients |
| --- | --- |
| Difficult left ventricular venting / myocardial distention | n = 3 |
| Relatively short anticipated AoX duration | n = 2 |
| Learning curve effect (early cases) | n = 4 |
| **Abbreviations:** AoX = aortic cross-clamp | |

**Supplementary Table S2.1: as-treated analyses for complete cohort (2014-2025), baseline characteristics**

| **Parameter** | Conventional (n=91) | Total body perfusion (n=30) | p-value |
| --- | --- | --- | --- |
| **Demographics** | | | |
| Male (n, %) | 50 (55%) | 19 (63%) | .554 |
| Age (years) | 65.5 [56.8-70.1] | 65.4 [61.9-73.7] | .248 |
| BMI (kg/m^2^) | 25.8 [23.3-29.0] | 25.6 [23.2-29.5] | .926 |
| EuroSCORE II | 4.8 [2.9-9.3] | 5.0 [3.5-7.0] | .875 |
| **Comorbidities and cardiac function** | | | |
| Diabetes (n, %) | 4 (4%) | 2 (7%) | .637 |
| Preoperative serum creatinine (umol/L) | 86 [73.0-104.0] | 87.5 [70.0-100.0] | .834 |
| Preoperative eGFR  (mean ± SD, ml/min) | 77.0 ± 19.0 | 78.1 ± 19.8 | .781 |
| LVEF (%) | 55 [52-58] | 55 [46.2-58.5] | .250 |
| **Operative characteristics** | | | |
| Reoperation (n, %) | 22 (24) | 5 (17%) | .546 |
| Chronic aortic arch dissection (n, %) | 1 (1%) | 6 (20%) | **<.001 *** |
| Active infective endocarditis, prosthetic infection, or mycotic aneurysm at time of surgery (n, %) | 1 (1%) | 2 (7%) | .152 |
| *All values are presented as median [IQR], unless stated otherwise.*  Abbreviations: BMI = Body Mass Index; eGFR = estimated glomerular filtration rate (as estimated by CKD-EPI); IQR = interquartile range; LVEF = left ventricular ejection fraction; SD = standard deviation | | | |

**Supplementary Table S2.2: as-treated analyses for complete cohort (2014-2025), intraoperative characteristics**

| Parameter | Conventional (n=91) | Total body perfusion (n=30) | p-value |
| --- | --- | --- | --- |
| **Concomitant procedures** | | | |
| Concomitant aortic root procedure (n, %) | 24 (26%) | 13 (43%) | .163 |
| Any concomitant cardiac surgery (n, %) | 42 (46%) | 21 (70%) | **.040 *** |
| Total arch replacement (n, %) | 52 (57%) | 27 (90%) | **.001*** |
| FET/ET (n, %) | 33 (36%) | 13 (43%) | .635 |
| **Operative parameters** | | | |
| Lowest temperature (°C) | 25.3 [24.6-28.0] | 29.4 [28.0-30.0] | **<.001 *** |
| ECC duration (minutes) | 228.0 [192.5-279.0] | 230.0 [216.0-276.0] | .628 |
| Myocardial ischemia time (minutes) | 116.0 [90.5-160.0] | 52.0 [35.0-90.0] | **<.001 *** |
| Circulatory arrest (n, %) | 76 (84%) | 3 (10%) | **<.001 *** |
| Circulatory arrest time (minutes) | 42.0 [22.0-55.0] | 0.0 [0.0-9.0] | **<.001 *** |
| *All values are presented as median [IQR], unless stated otherwise.*  Abbreviations: ECC = extracorporeal circulation; ET = elephant trunk; FET = frozen elephant trunk; IQR = interquartile range. | | | |

**Supplementary Table S2.3: as-treated analyses for complete cohort (2014-2025), biochemical outcomes**

| Parameter | Reference | Conventional (n=91) | Total body perfusion (n=30) | p-value |
| --- | --- | --- | --- | --- |
| **Peak lactate levels** | | | | |
| Intraoperatively (mmol/L) | 0.6-2.4 | 3.8 [2.8-5.0] | 2.2 [1.5-3.3] | **<.001 *** |
| 48h postoperatively (mmol/L) |  | 3.5 [2.1-6.2] | 2.4 [1.4-3.9] | **.018 *** |
| 48h postoperatively (including intra-operative values) (mmol/L) |  | 4.6 [3.2-6.5] | 2.8 [1.6-4.6] | **.002 *** |
| **Peak concentrations cardiac biomarkers 48h postoperatively** | | | | |
| CK (U/L) | <160 | 810.0 [485.0-1623.0] | 747.0 [466.2-1231.2] | .661 |
| CKMB (ug/L) | <2.9 | 40.2 [27.4-119.2] | 39.7 [30.4-80.2] | .692 |
| hsTnT (ng/L) | <14 | 1810.0 [375.0-4847.2] | 2011.0 [929.0-2740.0] | .896 |
| **Renal biomarkers** | | | | |
| Peak serum creatinine concentration 48h postoperatively (umol/L) | 50-100 | 108.0 [86.0-170.5] | 105.0 [87.0-130.0] | .597 |
| Maximum creatinine elevation from baseline |  | 20.0 [1.5-65.5] | 17.5 [4.5-47.0] | .499 |
| *All values are presented as median [IQR], unless stated otherwise.*  Abbreviations: CK = creatinine kinase; CK-MB = creatinine kinase-M; hsTnT = high-sensitivity troponin T; IQR = interquartile range. | | | | |

**Supplementary Table S2.4: as-treated analyses for complete cohort (2014-2025), clinical outcomes**

| Parameter **^1^** | Conventional (n=91) | Total body perfusion (n=30) | p-value |
| --- | --- | --- | --- |
| **Mortality** | | | |
| In-hospital mortality (n, %) | 18 (20%) | 3 (10%) | .276 |
| **Cardiovascular complications** | | | |
| Myocardial infarction (n, %) | 2 (2%) | 1 (3%) | 1.000 |
| **Respiratory complications** | | | |
| Pulmonary infection (n, %) | 13 (14%) | 8 (27%) | .202 |
| Respiratory insufficiency (n, %) | 13 (14%) | 2 (7%) | .390 |
| **Neurological complications** | | | |
| Permanent neurological dysfunction (n, %) | 11 (12%) | 1 (3%) | .290 |
| Transient neurological dysfunction (n, %) | 6 (7%) | 2 (7%) | 1.000 |
| Total stroke incidence (n, %) | 17 (19%) | 3 (10%) | .397 |
| **Renal and gastrointestinal complications** | | | |
| Acute renal failure (n, %) | 20 (22%) | 4 (13%) | .430 |
| Gastrointestinal complications (n, %) | 8 (9%) | 1 (3%) | .449 |
| **ICU management and surgical complications** | | | |
| ECMO (n, %) | 8 (9%) | 1 (3%) | .449 |
| ICU readmission (n, %) | 15 (16%) | 3 (10%) | .557 |
| Rethoracotomy (n, %) | 16 (18%) | 3 (10%) | .398 |
| Cardiac tamponade (n, %) | 5 (5%) | 2 (7%) | 1.000 |
| **Length of stay** | | | |
| ICU length of stay (days) | 4.0 [2.0-7.2] | 2.5 [1.0-7.0] | **.046*** |
| Hospital length of stay (days) | 9 [6.0-17.0] | 8.0 [6.0-15.0] | .533 |
| *All values are presented as median [IQR], unless stated otherwise.*  **Abbreviations:** ECMO = extracorporeal membrane oxygenation; ICU = intensive care unit; IQR = interquartile range.  ^1^ Detailed definitions of all outcomes are provided in the Supplementary Material (S1). | | | |

**Supplementary Table S3.1: per protocol analyses for complete cohort (2014-2025), baseline characteristics**

| **Parameter** | Conventional (n=83) | Total body perfusion (n=30) | p-value |
| --- | --- | --- | --- |
| **Demographics** | | | |
| Male (n, %) | 44 (53%) | 19 (63%) | .447 |
| Age (years) | 66.1 [58.7-70.6] | 65.4 [61.9-73.7] | .389 |
| BMI (kg/m^2^) | 25.8 [23.2-29.0] | 25.6 [23.2-29.5] | .977 |
| EuroSCORE II | 5.1 [3.0-9.5] | 5.0 [3.5-7.0] | .992 |
| **Comorbidities and cardiac function** | | | |
| Diabetes (n, %) | 3 (4%) | 2 (7%) | .607 |
| Preoperative serum creatinine (umol/L) | 85 [72.0-105.5] | 87.5 [70.0-100.0] | .822 |
| Preoperative eGFR  (mean ± SD, ml/min) | 76.3 ± 18.9 | 78.1 ± 19.8 | .675 |
| LVEF (%) | 55 [54-58] | 55 [46.2-58.5] | .232 |
| **Operative characteristics** | | | |
| Reoperation (n, %) | 19 (23%) | 5 (17%) | .650 |
| Chronic aortic arch dissection (n, %) | 0 (0%) | 6 (20%) | **<.001 *** |
| Active infective endocarditis, prosthetic infection, or mycotic aneurysm at time of surgery (n, %) | 1 (1%) | 2 (7%) | .172 |
| *All values are presented as median [IQR], unless stated otherwise.*  Abbreviations: BMI = Body Mass Index; eGFR = estimated glomerular filtration rate (as estimated by CKD-EPI); IQR = interquartile range; LVEF = left ventricular ejection fraction; SD = standard deviation | | | |

**Supplementary Table S3.2: per protocol analyses for complete cohort (2014-2025), intraoperative characteristics**

| Parameter | Conventional (n=83) | Total body perfusion (n=30) | p-value |
| --- | --- | --- | --- |
| **Concomitant procedures** | | | |
| Concomitant aortic root procedure (n, %) | 23 (28%) | 13 (43%) | .224 |
| Any concomitant cardiac surgery (n, %) | 38 (46%) | 21 (70%) | **.039 *** |
| Total arch replacement (n, %) | 49 (59%) | 27 (90%) | **.002*** |
| FET/ET (n, %) | 31 (37%) | 13 (43%) | .721 |
| **Operative parameters** | | | |
| Lowest temperature (°C) | 25.0 [24.6-28.0] | 29.4 [28.0-30.0] | **<.001 *** |
| ECC duration (minutes) | 228.0 [195.0-281.5] | 230.0 [216.0-276.0] | .775 |
| Myocardial ischemia time (minutes) | 122.0 [94.0-165.0] | 52.0 [35.0-90.0] | **<.001 *** |
| Circulatory arrest (n, %) | 72 (87%) | 3 (10%) | **<.001 *** |
| Circulatory arrest time (minutes) | 44.0 [26.5-55.0] | 0.0 [0.0-9.0] | **<.001 *** |
| *All values are presented as median [IQR], unless stated otherwise.*  Abbreviations: ECC = extracorporeal circulation; ET = elephant trunk; FET = frozen elephant trunk; IQR = interquartile range. | | | |

**Supplementary Table S3.3: per protocol analyses for complete cohort (2014-2025), biochemical outcomes**

| Parameter | Reference | Conventional (n=83) | Total body perfusion (n=30) | p-value |
| --- | --- | --- | --- | --- |
| **Peak lactate levels** | | | | |
| Intraoperatively (mmol/L) | 0.6-2.4 | 3.8 [3.0-5.0] | 2.2 [1.5-3.3] | **<.001 *** |
| 48h postoperatively (mmol/L) |  | 3.6 [2.3-6.3] | 2.4 [1.4-3.9 | **.016 *** |
| 48h postoperatively (including intra-operative values) (mmol/L) |  | 4.8 [3.4-6.8] | 2.8 [1.6-4.6] | **.001 *** |
| **Peak concentrations cardiac biomarkers 48h postoperatively** | | | | |
| CK (U/L) | <160 | 810.0 [491.0-1727.0] | 747.0 [466.2-1231.2] | .580 |
| CKMB (ug/L) | <2.9 | 41.1 [28.3-138.5] | 39.7 [30.4-80.2] | .485 |
| hsTnT (ng/L) | <14 | 2573.0 [823.0-8498.5] | 2011.0 [929.0-2740.0] | .640 |
| **Renal biomarkers** | | | | |
| Peak serum creatinine concentration 48h postoperatively (umol/L) | 50-100 | 111.0 [86.5-172.5] | 105.0 [87.0-130.0] | .443 |
| Maximum creatinine elevation from baseline |  | 22.0 [3.0-67.5] | 17.5 [4.5-47.0] | .361 |
| *All values are presented as median [IQR], unless stated otherwise.*  Abbreviations: CK = creatinine kinase; CK-MB = creatinine kinase-M; hsTnT = high-sensitivity troponin T; IQR = interquartile range. | | | | |

**Supplementary Table S3.4: per protocol analyses for complete cohort (2014-2025), clinical outcomes**

| Parameter | Conventional (n=83) | Total body perfusion (n=30) | p-value |
| --- | --- | --- | --- |
| **Mortality** | | | |
| In-hospital mortality (n, %) | 18 (22%) | 3 (10%) | .184 |
| **Cardiovascular complications** | | | |
| Myocardial infarction (n, %) | 2 (2%) | 1 (3%) | 1.000 |
| **Respiratory complications** | | | |
| Pulmonary infection (n, %) | 13 (16%) | 8 (27%) | .292 |
| Respiratory insufficiency (n, %) | 12 (14%) | 2 (7%) | .347 |
| **Neurological complications** | | | |
| Permanent neurological dysfunction (n, %) | 11 (13%) | 1 (3%) | .292 |
| Transient neurological dysfunction (n, %) | 5 (6.0%) | 2 (7%) | 1.000 |
| Total stroke incidence (n, %) | 16 (19%) | 3 (10%) | .393 |
| **Renal and gastrointestinal complications** | | | |
| Acute renal failure (n, %) | 19 (23%) | 4 (13%) | .305 |
| Gastrointestinal complications (n, %) | 8 (10%) | 1 (3%) | .441 |
| **ICU management and surgical complications** | | | |
| ECMO (n, %) | 7 (8%) | 1 (3%) | .679 |
| ICU readmission (n, %) | 14 (17%) | 3 (10%) | .553 |
| Rethoracotomy (n, %) | 15 (18%) | 3 (10%) | .391 |
| Cardiac tamponade (n, %) | 4 (5%) | 2 (7%) | .655 |
| **Length of stay** | | | |
| ICU length of stay (days) | 4.0 [3.0-8.0] | 2.5 [1.0-7.0] | **.028*** |
| Hospital length of stay (days) | 10.0 [6.0-17.0] | 8.0 [6.0-15.0] | .361 |
| *All values are presented as median [IQR], unless stated otherwise.*  **Abbreviations:** ECMO = extracorporeal membrane oxygenation; ICU = intensive care unit; IQR = interquartile range.  ^1^ Detailed definitions of all outcomes are provided in the Supplementary Material (S1). | | | |

**Supplementary Table S4.1.1: intention-to-treat analyses for complete TAR-cohort (2014-2025), baseline characteristics**

| Parameters | Conventional  (n=49) | Total body perfusion (n=30) | p-value |
| --- | --- | --- | --- |
| **Demographics** | | | |
| Male (n, %) | 25 (51%) | 19 (63.3%) | .403 |
| Age (years) | 63.3 [56.0-69.2] | 65.2 [60.6-73.7] | .317 |
| BMI (kg/m^2^) | 25.5 [22.6-27.7] | 25.5 [23.2-29.5] | .551 |
| EuroSCORE II | 6.5 [3.4-9.7] | 5.0 [3.2-7.2] | .382 |
| **Comorbidities and cardiac function** | | | |
| Diabetes (n, %) | 0 (0.0%) | 2 (6.7%) | .141 |
| Preoperative serum creatinine (umol/L) | 83.0 [69-108] | 87.5 [73.5-96.2] | .932 |
| Preoperative eGFR  (mean ± SD, ml/min) | 77.9 ± 19.9 | 79.3 ± 19.0 | .756 |
| LVEF (%) | 55 [51-60] | 52.5 [46.2-56.8] | .112 |
| **Operative characteristics** | | | |
| Reoperation (n, %) | 13 (27%) | 6 (20%) | .698 |
| Chronic aortic arch dissection (n, %) | 0 (0.0%) | 7 (23%) | **<.001 *** |
| Active infective endocarditis, prosthetic infection, or mycotic aneurysm at time of surgery (n, %) | 1 (2%) | 2 (7%) | .554 |
| *All values are presented as median [IQR], unless stated otherwise.*  Abbreviations: BMI = Body Mass Index; eGFR = estimated glomerular filtration rate (as estimated by CKD-EPI); IQR = interquartile range; LVEF = left ventricular ejection fraction; SD = standard deviation; TAR = total arch replacement | | | |

**Supplementary Table S4.1.2: intention-to-treat analyses for complete TAR-cohort (2014-2025), intraoperative characteristics**

| Parameter | Conventional (n=49) | Total body perfusion (n=30) | p-value |
| --- | --- | --- | --- |
| **Concomitant procedures** | | | |
| Concomitant aortic root procedure (n, %) | 13 (27%) | 13 (43%) | .485 |
| Any concomitant cardiac surgery (n, %) | 21 (43%) | 18 (60%) | .212 |
| FET (n, %) | 31 (63%) | 15 (50%) | .355 |
| **Operative parameters** | | | |
| Lowest temperature (°C) | 25.0 [24.9-26.9] | 29.4 [28.0-30.0] | **<.001 *** |
| ECC duration (minutes) | 251.0 [225.0-339.0] | 230.0 [216.0-266.0] | **0.034*** |
| Myocardial ischemia time (minutes) | 135.0 [93.0-195.0] | 52.0 [35.0-81.0] | **<.001 *** |
| Circulatory arrest (n, %) | 48 (98%) | 5 (17%) | **<.001 *** |
| Circulatory arrest time (minutes) | 48.0 [40.0-63.0] | 0.0 [0.0-18.0] | **<.001 *** |
| *All values are presented as median [IQR], unless stated otherwise.*  Abbreviations: ECC = extracorporeal circulation; ET = elephant trunk; FET = frozen elephant trunk; IQR = interquartile range; TAR = total arch replacement | | | |

**Supplementary Table S4.1.3: intention-to-treat analyses for complete TAR-cohort (2014-2025), biochemical outcomes**

| Parameter | Reference | Conventional (n=49) | Total body perfusion (n=30) | p-value |
| --- | --- | --- | --- | --- |
| **Peak lactate levels** | | | | |
| Intraoperatively (mmol/L) | 0.6-2.4 | 4.3 [3.1-5.8] | 2.3 [1.5-4.3] | **<.001 *** |
| 48h postoperatively (mmol/L) |  | 4.0 [2.4-7.0] | 2.7 [1.4-4.0] | .053 |
| 48h postoperatively (including intra-operative values) (mmol/L) |  | 5.0 [3.9-7.3] | 3.1 [1.6-5.1] | **.003** |
| **Peak concentrations cardiac biomarkers 48h postoperatively** | | | | |
| CK (U/L) | <160 | 1113.0 [583.0-2563.0] | 747.0 [466.2-1395.5] | .066 |
| CKMB (ug/L) | <2.9 | 41.1 [32.8-228.6] | 39.7 [30.4-80.2] | .180 |
| hsTnT (ng/L) | <14 | 3487.5 [616.5-9878.2] | 2084.5 [959.0-2785.0] | .670 |
| **Renal biomarkers** | | | | |
| Peak serum creatinine concentration 48h postoperatively (umol/L) | 50-100 | 131.0 [98.0-198.0] | 104.0 [87.0-138.0] | .071 |
| Maximum creatinine elevation from baseline |  | 45.0 [14.0-107.0] | 19.0 [6.0-47.8] | **.048*** |
| *All values are presented as median [IQR], unless stated otherwise.*  Abbreviations: CK = creatinine kinase; CK-MB = creatinine kinase-M; hsTnT = high-sensitivity troponin T; IQR = interquartile range; TAR = total arch replacement | | | | |

**Supplementary Table S4.1.4: intention-to-treat analyses for complete TAR-cohort (2014-2025), clinical outcomes**

| Parameter | Conventional (n=49) | Total body perfusion (n=30) | p-value |
| --- | --- | --- | --- |
| **Mortality** | | | |
| In-hospital mortality (n, %) | 14 (28.6%) | 3 (10%) | .095 |
| **Cardiovascular complications** | | | |
| Myocardial infarction (n, %) | 1 (2.0%) | 1 (3.3%) | 1.000 |
| **Respiratory complications** | | | |
| Pulmonary infection (n, %) | 9 (18.4%) | 7 (23.3%) | .807 |
| Respiratory insufficiency (n, %) | 9 (18.4%) | 2 (6.7%) | .191 |
| **Neurological complications** | | | |
| Permanent neurological dysfunction (n, %) | 7 (14.3%) | 1 (3.3%) | .147 |
| Transient neurological dysfunction (n, %) | 4 (8.2%) | 3 (10.0%) | 1.000 |
| Total stroke incidence (n, %) | 11 (22.4%) | 4 (13,3%) | .480 |
| **Renal and gastrointestinal complications** | | | |
| Acute renal failure (n, %) | 16 (32.7%) | 5 (16.7%) | .194 |
| Gastrointestinal complications (n, %) | 7 (14.3%) | 1 (3,3%) | .147 |
| **ICU management and surgical complications** | | | |
| ECMO (n, %) | 7 (14.3%) | 2 ?(6.7%) | .470 |
| ICU readmission (n, %) | 10 (20.4%) | 3 (10.0%) | .350 |
| Rethoracotomy (n, %) | 10 (20.4%) | 3 (10.0%) | .350 |
| Cardiac tamponade (n, %) | 3 (6.1%) | 2 (6.7%) | 1.000 |
| **Length of stay** | | | |
| ICU length of stay (days) | 5.0 [3.0-9.0] | 2.5 [1.0-7.0] | **.016*** |
| Hospital length of stay (days) | 13.0 [7.0-29.0] | 8.0 [6.0-15.0] | .109 |
| *All values are presented as median [IQR], unless stated otherwise.*  **Abbreviations:** ECMO = extracorporeal membrane oxygenation; ICU = intensive care unit; IQR = interquartile range; TAR = total arch replacement  ^1^ Detailed definitions of all outcomes are provided in the Supplementary Material (S1). | | | |

**Supplementary Table S4.2.1: as-treated analyses for complete TAR-cohort (2014-2025), baseline characteristics**

| Parameters | Conventional  (n=52) | Total body perfusion (n=27) | p-value |
| --- | --- | --- | --- |
| **Demographics** | | | |
| Male (n, %) | 27 (51.9%) | 17 (63.0%) | .485 |
| Age (years) | 62.9 [55.3-68.9] | 65.5 [61.9-74.0] | .098 |
| BMI (kg/m^2^) | 25.6 [22.8-27.7] | 25.3[ [22.8-29.2] | .836 |
| EuroSCORE II | 6.1 [3.3-9.7] | 5.2 [3.3-8.5] | .599 |
| **Comorbidities and cardiac function** | | | |
| Diabetes (n, %) | 0 (0.0%) | 2 (7.4%) | .114 |
| Preoperative serum creatinine (umol/L) | 83.5 [69-106.5] | 87.0 [70.5-95.5] | .780 |
| Preoperative eGFR  (mean ± SD, ml/min) | 77.9 ± 19.4 | 79.4 ± 19.9 | .743 |
| LVEF (%) | 55.0 (50.0-58.5] | 54.0 [45.5-58.0] | .257 |
| **Operative characteristics** | | | |
| Reoperation (n, %) | 15 (28.8%) | 4 (14.8%) | .269 |
| Chronic aortic arch dissection (n, %) | 1 (1.9%) | 6 (22.2%) | **.002*** |
| Active infective endocarditis, prosthetic infection, or mycotic aneurysm at time of surgery (n, %) | 1 (1.9%) | 2 (7.4%) | .268 |
| *All values are presented as median [IQR], unless stated otherwise.*  Abbreviations: BMI = Body Mass Index; eGFR = estimated glomerular filtration rate (as estimated by CKD-EPI); IQR = interquartile range; LVEF = left ventricular ejection fraction; SD = standard deviation; TAR = total arch replacement | | | |

**Supplementary Table S4.2.2: as-treated analyses for complete TAR-cohort (2014-2025), intraoperative characteristics**

| Parameter | Conventional (n=52) | Total body perfusion (n=27) | p-value |
| --- | --- | --- | --- |
| **Concomitant procedures** | | | |
| Concomitant aortic root procedure (n, %) | 13 (25%) | 11 (40.7%) | .236 |
| Any concomitant cardiac surgery (n, %) | 21 (40.4%) | 18 (66.7) | **.048*** |
| FET (n, %) | 33 (63.5%) | 12 (48.1%) | .285 |
| **Operative parameters** | | | |
| Lowest temperature (°C) | 25.0 [25.0-27.1] | 29.7 [28.0-30.0] | **<.001 *** |
| ECC duration (minutes) | 253.5 [225.8-315.8] | 228.5 [210.0-265.5] | **0.023*** |
| Myocardial ischemia time (minutes) | 129.0 [90.8-189.0] | 52.0 [29.9-79.8] | **<.001 *** |
| Circulatory arrest (n, %) | 50 (96.2%) | 3 (11.1%) | **<.001 *** |
| Circulatory arrest time (minutes) | 48.0 [39.5-63.0] | 0.0 [0.0-12.8] | **<.001 *** |
| *All values are presented as median [IQR], unless stated otherwise.*  Abbreviations: ECC = extracorporeal circulation; ET = elephant trunk; FET = frozen elephant trunk; IQR = interquartile range; TAR = total arch replacement | | | |

**Supplementary Table S4.2.3: as-treated analyses for complete TAR-cohort (2014-2025), biochemical outcomes**

| Parameter | Reference | Conventional (n=52) | Total body perfusion (n=27) | p-value |
| --- | --- | --- | --- | --- |
| **Peak lactate levels** | | | | |
| Intraoperatively (mmol/L) | 0.6-2.4 | 4.5 [3.2-5.9] | 2.1 [1.4-3.3] | **<.001 *** |
| 48h postoperatively (mmol/L) |  | 5.0 [4.1-7.4] | 2.8 [1.5-4.6] | **<.001*** |
| 48h postoperatively (including intra-operative values) (mmol/L) |  | 3.9 [2.4-7.0] | 2.7 [1.4-4.0] | **.035*** |
| **Peak concentrations cardiac biomarkers 48h postoperatively** | | | | |
| CK (U/L) | <160 | 1059.5 [582.0-2588.8] | 702.0 [484.5-1334.0] | .077 |
| CKMB (ug/L) | <2.9 | 40.6 [29.2-196.9] | 39.9 [32.4-77.9] | .396 |
| hsTnT (ng/L) | <14 | 2893.0 [375.0-7118.8] | 2084.5 [1014.2-2700.0] | .912 |
| **Renal biomarkers** | | | | |
| Peak serum creatinine concentration 48h postoperatively (umol/L) | 50-100 | 120.5 [96.5-200.0] | 102.5 [88.0-136.0] | .084 |
| Maximum creatinine elevation from baseline |  | 90.5 [73.2-106.2] | 80.5 [59.5-94.8] | .091 |
| *All values are presented as median [IQR], unless stated otherwise.*  Abbreviations: CK = creatinine kinase; CK-MB = creatinine kinase-M; hsTnT = high-sensitivity troponin T; IQR = interquartile range; TAR = total arch replacement | | | | |

**Supplementary Table S4.2.4: as-treated analyses for complete TAR-cohort (2014-2025), clinical outcomes**

| Parameter | Conventional (n=52) | Total body perfusion (n=27) | p-value |
| --- | --- | --- | --- |
| **Mortality** | | | |
| In-hospital mortality (n, %) | 14 (26.9%) | 3 (11.1%) | .182 |
| **Cardiovascular complications** | | | |
| Myocardial infarction (n, %) | 1 (1.9%) | 1 (3.7%) | 1.000 |
| **Respiratory complications** | | | |
| Pulmonary infection (n, %) | 9 (17.3%) | 7 (25.9%) | 1.000 |
| Respiratory insufficiency (n, %) | 9 (17.3%) | 2 (7.4%) | .315 |
| **Neurological complications** | | | |
| Permanent neurological dysfunction (n, %) | 7 (13.5%) | 1 (3.7%) | .253 |
| Transient neurological dysfunction (n, %) | 5 (9.6%) | 2 (7.4%) | .315 |
| Total stroke incidence (n, %) | 12 (23.1%) | 3 (11.1%) | .325 |
| **Renal and gastrointestinal complications** | | | |
| Acute renal failure (n, %) | 17 (32.7%) | 4 (14.8%) | .151 |
| Gastrointestinal complications (n, %) | 7 (13.5%) | 1 (3.7%) | .253 |
| **ICU management and surgical complications** | | | |
| ECMO (n, %) | 8 (15.4%) | 1 (3.7%) | .155 |
| ICU readmission (n, %) | 10 (19.2%) | 3 (11.1%) | .525 |
| Rethoracotomy (n, %) | 10 (19.2%) | 3 (11.1%) | .525 |
| Cardiac tamponade (n, %) | 5 (9.6%) | 3 (11.1%) | 1.000 |
| **Length of stay** | | | |
| ICU length of stay (days) | 5.0 [3.0-9.0] | 2.5 [1.0-7.0] | **.016*** |
| Hospital length of stay (days) | 13.0 [7.0-29.0] | 8.0 [6.0-15.0] | .109 |
| *All values are presented as median [IQR], unless stated otherwise.*  **Abbreviations:** ECMO = extracorporeal membrane oxygenation; ICU = intensive care unit; IQR = interquartile range; TAR = total arch replacement  ^1^ Detailed definitions of all outcomes are provided in the Supplementary Material (S1). | | | |

**Supplementary Table S4.3.1: per protocol analyses for complete TAR-cohort (2014-2025), baseline characteristics**

| Parameters | Conventional  (n=49) | Total body perfusion (n=27) | p-value |
| --- | --- | --- | --- |
| **Demographics** | | | |
| Male (n, %) | 24 (49.0%) | 10 (37.0%) | .447 |
| Age (years) | 63.3 [56.0-69.2] | 65.5 [61.9-74.0] | .147 |
| BMI (kg/m^2^) | 25.5 [22.6-27.7] | 25.3 [22.8-29.2] | .716 |
| EuroSCORE II | 6.5 [3.4-9.7] | 5.2 [2.2-8.5] | .526 |
| **Comorbidities and cardiac function** | | | |
| Diabetes (n, %) | 0 (0.0%) | 2 (7.4%) | .123 |
| Preoperative serum creatinine (umol/L) | 83.0 [69.0-108.0] | 87.0 [70.5-95.5] | .832 |
| Preoperative eGFR  (mean ± SD, ml/min) | 77.9 ± 19.9 | 79.4 ± 19.9 | .747 |
| LVEF (%) | 55.0 [51.0-60.0] | 54.0 [45.5-58.0] | .200 |
| **Operative characteristics** | | | |
| Reoperation (n, %) | 13 (26.5%) | 4 (14.8%) | .376 |
| Chronic aortic arch dissection (n, %) | 0 (0%) | 6 (22.2%) | **<.001*** |
| Active infective endocarditis, prosthetic infection, or mycotic aneurysm at time of surgery (n, %) | 1 (2.0%) | 2 (7.4%) | .286 |
| *All values are presented as median [IQR], unless stated otherwise.*  Abbreviations: BMI = Body Mass Index; eGFR = estimated glomerular filtration rate (as estimated by CKD-EPI); IQR = interquartile range; LVEF = left ventricular ejection fraction; SD = standard deviation; TAR = total arch replacement | | | |

**Supplementary Table S4.3.2: per protocol analyses for complete TAR-cohort (2014-2025), intraoperative characteristics**

| Parameter | Conventional (n=49) | Total body perfusion (n=27) | p-value |
| --- | --- | --- | --- |
| **Concomitant procedures** | | | |
| Concomitant aortic root procedure (n, %) | 13 (26.5%) | 11 (40.7%) | .308 |
| Any concomitant cardiac surgery (n, %) | 21 (42.9%) | 18 (66.7%) | .081 |
| FET (n, %) | 31 (63.3%) | 13 (48.1%) | .301 |
| **Operative parameters** | | | |
| Lowest temperature (°C) | 25.0 [24.9-26.9] | 29.7 [28.0-30.0] | **<.001 *** |
| ECC duration (minutes) | 251.0 [225.0-339.0] | 228.5 [210.0-265.5] | **0.025*** |
| Myocardial ischemia time (minutes) | 135.0 [93.0-195.0] | 52.0 [29.8-79.8] | **<.001 *** |
| Circulatory arrest (n, %) | 48 (98%) | 3 (11.1%) | **<.001 *** |
| Circulatory arrest time (minutes) | 48.0 [40.0-63.0] | 0.0 [0.0-12.8] | **<.001 *** |
| *All values are presented as median [IQR], unless stated otherwise.*  Abbreviations: ECC = extracorporeal circulation; ET = elephant trunk; FET = frozen elephant trunk; IQR = interquartile range; TAR = total arch replacement | | | |

**Supplementary Table S4.3.3: per protocol analyses for complete TAR-cohort (2014-2025), biochemical outcomes**

| Parameter | Reference | Conventional (n=49) | Total body perfusion (n=27) | p-value |
| --- | --- | --- | --- | --- |
| **Peak lactate levels** | | | | |
| Intraoperatively (mmol/L) | 0.6-2.4 | 4.3 [3.1-5.8] | 2.1 [1.4-3.3] | **<.001 *** |
| 48h postoperatively (mmol/L) |  | 4.0 [2.4-7.0] | 2.7 [1.4-4.0] | **<.001*** |
| 48h postoperatively (including intra-operative values) (mmol/L) |  | 5.0 [3.9-7.3] | 2.8 [1.5-4.5] | **<.001*** |
| **Peak concentrations cardiac biomarkers 48h postoperatively** | | | | |
| CK (U/L) | <160 | 1113.0 [583.0-2563.0] | 702.0 [484.5-1334.0] | .067 |
| CKMB (ug/L) | <2.9 | 41.1 [32.8-228.6] | 39.9 [32.4-77.9] | .288 |
| hsTnT (ng/L) | <14 | 3487.5 [616.5-9879.2] | 2084.5 [1014.2-2700.0] | .790 |
| **Renal biomarkers** | | | | |
| Peak serum creatinine concentration 48h postoperatively (umol/L) | 50-100 | 131.0 [98.0-198.0] | 102.5 [88.0-136.0] | .073 |
| Maximum creatinine elevation from baseline |  | 45.0 [14.0-107.0] | 18.0 [16.0-47.5] | .054 |
| *All values are presented as median [IQR], unless stated otherwise.*  Abbreviations: CK = creatinine kinase; CK-MB = creatinine kinase-M; hsTnT = high-sensitivity troponin T; IQR = interquartile range; TAR = total arch replacement | | | | |

**Supplementary Table S4.3.4: per protocol analyses for complete TAR-cohort (2014-2025), clinical outcomes**

| Parameter | Conventional (n=49) | Total body perfusion (n=27) | p-value |
| --- | --- | --- | --- |
| **Mortality** | | | |
| In-hospital mortality (n, %) | 14 (28.6%) | 3 (11.1%) | .144 |
| **Cardiovascular complications** | | | |
| Myocardial infarction (n, %) | 1 (2.0%) | 1 (3.7%) | 1.000 |
| **Respiratory complications** | | | |
| Pulmonary infection (n, %) | 9 (18.4%) | 7 (25.9%) | .632 |
| Respiratory insufficiency (n, %) | 9 (18.4%) | 2 (7.4%) | .309 |
| **Neurological complications** | | | |
| Permanent neurological dysfunction (n, %) | 7 (14.3%) | 1 (3.7%) | .247 |
| Transient neurological dysfunction (n, %) | 4 (8.2%) | 2 (7.4%) | 1.000 |
| Total stroke incidence (n, %) | 11 (22.4%) | 3 (11.1%) | .355 |
| **Renal and gastrointestinal complications** | | | |
| Acute renal failure (n, %) | 16 (32.7%) | 4 (14.8%) | .156 |
| Gastrointestinal complications (n, %) | 7 (14.3%) | 1 (3.7%) | .247 |
| **ICU management and surgical complications** | | | |
| ECMO (n, %) | 7 (14.3%) | 7 (14.3%) | .247 |
| ICU readmission (n, %) | 10 (20.4%) | 3 (11.1%) | .359 |
| Rethoracotomy (n, %) | 10 (20.4%) | 3 (11.1%) | .359 |
| Cardiac tamponade (n, %) | 3 (6.1%) | 2 (7.4%) | 1.000 |
| **Length of stay** | | | |
| ICU length of stay (days) | 5.0 [3.0-9.0] | 2.0 [1.0-7.0] | **.008*** |
| Hospital length of stay (days) | 13.0 [7.0-29.0] | 8.0 [6.0-15.0] | .098 |
| *All values are presented as median [IQR], unless stated otherwise.*  **Abbreviations:** ECMO = extracorporeal membrane oxygenation; ICU = intensive care unit; IQR = interquartile range; TAR = total arch replacement  ^1^ Detailed definitions of all outcomes are provided in the Supplementary Material (S1). | | | |

**Supplementary Table S5.1.1: intention-to-treat analyses for complete cohort (2020-2025), baseline characteristics**

| **Parameter** | Conventional (n=15) | Total body perfusion (n=38) | p-value |
| --- | --- | --- | --- |
| **Demographics** | | | |
| Male (n, %) | 11 (73.3%) | 25 (65.8%) | .748 |
| Age (years) | 63.3 [57.4-67.8] | 64.5 [60.6-72.3] | .418 |
| BMI (kg/m^2^) | 25.5 [23.0-27.4] | 25.7 [23.7-29.5] | .407 |
| EuroSCORE II | 5.6 [3.4-10.5] | 4.9 [3.2-6.4] | .478 |
| **Comorbidities and cardiac function** | | | |
| Diabetes (n, %) | 1 (6.7%) | 3 (7.9%) | 1.000 |
| Preoperative serum creatinine (umol/L) | 96.0 [77.5-105.0] | 87.5 [73.5-100.0] | .379 |
| Preoperative eGFR  (mean ± SD, ml/min) | 76.8 ± 24.7 | 79.2 ± 19.7 | .739 |
| LVEF (%) | 55 [53-55] | 55 [48.2-58.5] | .920 |
| **Operative characteristics** | | | |
| Reoperation (n, %) | 6 (40%) | 8 (21.1%) | 0.182 |
| Chronic aortic arch dissection (n, %) | 0 (0%) | 7 (18.4%) | .179 |
| Active infective endocarditis, prosthetic infection, or mycotic aneurysm at time of surgery (n, %) | 0 (0%) | 2 (5.3%) | 1.000 |
| *All values are presented as median [IQR], unless stated otherwise.*  Abbreviations: BMI = Body Mass Index; eGFR = estimated glomerular filtration rate (as estimated by CKD-EPI); IQR = interquartile range; LVEF = left ventricular ejection fraction; SD = standard deviation | | | |

**Supplementary Table S5.1.2: intention-to-treat analyses for complete cohort (2020-2025), intraoperative characteristics**

| Parameter | Conventional (n=15) | Total body perfusion (n=38) | p-value |
| --- | --- | --- | --- |
| **Concomitant procedures** | | | |
| Concomitant aortic root procedure (n, %) | 3 (20%) | 15 (39.5%) | .333 |
| Any concomitant cardiac surgery (n, %) | 5 (33.3%) | 25 (65.8%) | .066 |
| Total arch replacement (, %) | 11 (73.3%) | 30 (78.9%) | .722 |
| FET/ET (n, %) | 9 (60.0%) | 15 (39.5%) | .296 |
| **Operative parameters** | | | |
| Lowest temperature (°C) | 26.0 [25.1-27.2] | 29.8 [28.0-31.7] | **<.001*** |
| ECC duration (minutes)  (mean ± SD minutes) | 258.9 ± 58.2 | 233.2 ± 50.5 | 0.149 |
| Myocardial ischemia time (mean ± SD minutes) | 123.7 ± 52.1 | 67.3 ± 41.1 | **.001*** |
| Circulatory arrest (n, %) | 13 (86.7%) | 7 (18.4%) | **<.001*** |
| Circulatory arrest time (minutes) | 46.0 [38.5-55.0] | 0.0 [0.0-14.0] | **<.001*** |
| *All values are presented as median [IQR], unless stated otherwise.*  Abbreviations: ECC = extracorporeal circulation; ET = elephant trunk; FET = frozen elephant trunk; IQR = interquartile range. | | | |

**Supplementary Table S5.1.3: intention-to-treat analyses for complete cohort (2020-2025), biochemical outcomes**

| Parameter | Reference | Conventional (n=15) | Total body perfusion (n=38) | p-value |
| --- | --- | --- | --- | --- |
| **Peak lactate levels** | | | | |
| Intraoperatively (mmol/L) | 0.6-2.4 | 4.3 [3.0-4.8] | 2.2 [1.5-4.3] | **.019*** |
| 48h postoperatively (mmol/L) |  | 3.8 [2.0-6.3] | 2.7 [1.5-3.9] | .154 |
| 48h postoperatively (including intra-operative values) (mmol/L) |  | 4.3 [3.0-6.1] | 2.9 [1.8-5.1] | .078 |
| **Peak concentrations cardiac biomarkers 48h postoperatively** | | | | |
| CK (U/L) | <160 | 943.0 [670.0- 2875.0] | 800.0 [416.5-1193.5] | .179 |
| CKMB (ug/L) | <2.9 | 45.5 [31.1-287.0] | 38.8 [27.6,72.0] | .256 |
| hsTnT (ng/L) | <14 | 5739.0 [5739.0-5739.0] | 1741.5 [878.0-2700.0] | .158 |
| **Renal biomarkers** | | | | |
| Peak serum creatinine concentration 48h postoperatively (umol/L) | 50-100 | 183.0 [85.5-224.5] | 103.0 [86.0-128.0] | .166 |
| Maximum creatinine elevation from baseline |  | 63.0 [5.0-118.5] | 16.0 [-3.8-44.5] | .074 |
| *All values are presented as median [IQR], unless stated otherwise.*  Abbreviations: CK = creatinine kinase; CK-MB = creatinine kinase-M; hsTnT = high-sensitivity troponin T; IQR = interquartile range. | | | | |

**Supplementary Table S5.1.4: intention-to-treat analyses for complete cohort (2020-2025), clinical outcomes**

| Parameter **^1^** | Conventional (n=15) | Total body perfusion (n=38) | p-value |
| --- | --- | --- | --- |
| **Mortality** | | | |
| In-hospital mortality (n, %) | 7 (46.7%) | 3 (7.9%) | **.003*** |
| **Cardiovascular complications** | | | |
| Myocardial infarction (n, %) | 1 (6.7%) | 1 (2.6%) | .490 |
| **Respiratory complications** | | | |
| Pulmonary infection (n, %) | 1 (6.7%) | 8 (21.1%) | .418 |
| Respiratory insufficiency (n, %) | 3 (20.0%) | 3 (7.9) | .334 |
| **Neurological complications** | | | |
| Permanent neurological dysfunction (n, %) | 1 (6.7%) | 1 (2.6%) | .490 |
| Transient neurological dysfunction (n, %) | 1 (6.7%) | 3 (7.9%) | 1.000 |
| Total stroke incidence (n, %) | 2 (13.3%) | 4 (10.5%) | 1.000 |
| **Renal and gastrointestinal complications** | | | |
| Acute renal failure (n, %) | 7 (46.7%) | 5 (13.2%) | **.024*** |
| Gastrointestinal complications (n, %) | 3 (20.0%) | 1 (2.6%) | .064 |
| **ICU management and surgical complications** | | | |
| ECMO (n, %) | 1 (6.7%) | 2 (5.3%) | 1.000 |
| ICU readmission (n, %) | 3 (20.0%) | 4 (10.5%) | .389 |
| Rethoracotomy (n, %) | 3 (20.0%) | 4 (10.5%) | .389 |
| Cardiac tamponade (n, %) | 0 (0.0%) | 3 (7.9%) | .550 |
| **Length of stay** | | | |
| ICU length of stay (days) | 4.0 [2.5-4.5] | 2.0 [1.0-7.0] | .120 |
| Hospital length of stay (days) | 8.0 [3.5-12.0] | 8.0 [6.0-15.0] | .500 |
| *All values are presented as median [IQR], unless stated otherwise.*  **Abbreviations:** ECMO = extracorporeal membrane oxygenation; ICU = intensive care unit; IQR = interquartile range.  ^1^ Detailed definitions of all outcomes are provided in the Supplementary Material (S1). | | | |

**Supplementary Table S5.2.1: as-treated analyses for complete cohort (2020-2025), baseline characteristics**

| **Parameter** | Conventional (n=23) | Total body perfusion (n=30) | p-value |
| --- | --- | --- | --- |
| **Demographics** | | | |
| Male (n, %) | 17 (73.9%) | 19 (63.3%) | .602 |
| Age (years) | 61.5 [52.7-67.2] | 65.4 [61.9-73.7] | **.030*** |
| BMI (kg/m^2^) | 25.7 [23.8-27.8]] | 25.6 [23.2-29.5] | .737 |
| EuroSCORE II | 4.9 [2.7-6.7] | 5.0 [3.5-7.0] | .823 |
| **Comorbidities and cardiac function** | | | |
| Diabetes (n, %) | 2 (8.7%) | 2 (6.7%) | 1.000 |
| Preoperative serum creatinine (umol/L) | 90.0 [82.0-101.5] | 87.5 [70.0-100.0] | .512 |
| Preoperative eGFR  (mean ± SD, ml/min) | 79.0 ± 22.9 | 78.1 ± 19.8 | .880 |
| LVEF (%) | 55 [50.5-55.0] | 55.0 [46.2-58.5] | .834 |
| **Operative characteristics** | | | |
| Reoperation (n, %) | 9 (39.1%) | 5 (16.7%) | .127 |
| Chronic aortic arch dissection (n, %) | 1 (4.3%) | 6 (20.0%) | .123 |
| Active infective endocarditis, prosthetic infection, or mycotic aneurysm at time of surgery (n, %) | 0 (0%) | 2 (6.7%) | .499 |
| *All values are presented as median [IQR], unless stated otherwise.*  Abbreviations: BMI = Body Mass Index; eGFR = estimated glomerular filtration rate (as estimated by CKD-EPI); IQR = interquartile range; LVEF = left ventricular ejection fraction; SD = standard deviation | | | |

**Supplementary Table S5.2.2: as-treated analyses for complete cohort (2020-2025), intraoperative characteristics**

| Parameter | Conventional (n=23) | Total body perfusion (n=30) | p-value |
| --- | --- | --- | --- |
| **Concomitant procedures** | | | |
| Concomitant aortic root procedure (n, %) | 5 (21.7%) | 13 (43.3%) | .074 |
| Any concomitant cardiac surgery (n, %) | 9 (39.1%) | 21 (70.0%) | **.049*** |
| Total arch replacement (n, %) | 14 (60.9%) | 27 (90.0%) | **.029*** |
| FET/ET (n, %) | 11 (47.8%) | 12 (43.3%) | .962 |
| **Operative parameters** | | | |
| Lowest temperature (°C) | 27.1 [25.6-28.0] | 29.4 [28.0-30.0] | **.003*** |
| ECC duration  (mean ± SD minutes) | 240.8 ± 59.6 | 240.4 ± 49.3 | .981 |
| Myocardial ischemia time  (mean ± SD minutes) | 113.2 ± 45.9 | 60.0 ± 42.3 | **<.001*** |
| Circulatory arrest (n, %) | 17 (73.9%) | 3 (10.0%) | **<.001*** |
| Circulatory arrest time (minutes) | 42.0 [0.0-52.5] | 0.0 [0.0-9.0] | **.001*** |
| *All values are presented as median [IQR], unless stated otherwise.*  Abbreviations: ECC = extracorporeal circulation; ET = elephant trunk; FET = frozen elephant trunk; IQR = interquartile range. | | | |

**Supplementary Table S5.2.3: as-treated analyses for complete cohort (2020-2025), biochemical outcomes**

| Parameter | Reference | Conventional (n=23) | Total body perfusion (n=30) | p-value |
| --- | --- | --- | --- | --- |
| **Peak lactate levels** | | | | |
| Intraoperatively (mmol/L) | 0.6-2.4 | 4.3 [2.5-4.9] | 2.2 [1.5-3.3] | **.018*** |
| 48h postoperatively (mmol/L) |  | 3.5 [1.8-6.0] | 2.4 [1.4-3.9] | .119 |
| 48h postoperatively (including intra-operative values) (mmol/L) |  | 4.3 [3.0-5.6] | 2.8 [1.6-4.6] | **.042*** |
| **Peak concentrations cardiac biomarkers 48h postoperatively** | | | | |
| CK (U/L) | <160 | 838.0 [550.0-2255.0] | 747.0 [466.2-1231.2] | .495 |
| CKMB (ug/L) | <2.9 | 38.7 [27.6-110.7] | 39.7 [30.4-80.2] | .907 |
| hsTnT (ng/L) | <14 | 1810.0 [514.5-3983.0] | 2011.0 [929.0-2740.0] | .771 |
| **Renal biomarkers** | | | | |
| Peak serum creatinine concentration 48h postoperatively (umol/L) | 50-100 | 104.0 [84.5-210.0] | 105.0 [87.0-130.0] | .665 |
| Maximum creatinine elevation from baseline |  | 21.0 [-1.0-111.0] | 17.5 [4.5-47.0] | .548 |
| *All values are presented as median [IQR], unless stated otherwise.*  Abbreviations: CK = creatinine kinase; CK-MB = creatinine kinase-M; hsTnT = high-sensitivity troponin T; IQR = interquartile range. | | | | |

**Supplementary Table S5.2.4: as-treated analyses for complete cohort (2020-2025), clinical outcomes**

| Parameter **^1^** | Conventional (n=23) | Total body perfusion (n=30) | p-value |
| --- | --- | --- | --- |
| **Mortality** | | | |
| In-hospital mortality (n, %) | 7 (30.4%) | 3 (10.0%) | .082 |
| **Cardiovascular complications** | | | |
| Myocardial infarction (n, %) | 1 (4.3%) | 1 (3.3%) | 1.000 |
| **Respiratory complications** | | | |
| Pulmonary infection (n, %) | 1 (4.3%) | 8 (26.7%) | .061 |
| Respiratory insufficiency (n, %) | 4 (17.4%) | 2 (6.7%) | .385 |
| **Neurological complications** | | | |
| Permanent neurological dysfunction (n, %) | 1 (4.3%) | 1 (3.3%) | 1.000 |
| Transient neurological dysfunction (n, %) | 2 (8.7%) | 2 (6.7%) | 1.000 |
| Total stroke incidence (n, %) | 3 (13.0%) | 3 (10.0%) | 1.000 |
| **Renal and gastrointestinal complications** | | | |
| Acute renal failure (n, %) | 15 (65.2%) | 26 (86.7%) | .129 |
| Gastrointestinal complications (n, %) | 3 (13.0%) | 1 (3.3%) | .305 |
| **ICU management and surgical complications** | | | |
| ECMO (n, %) | 2 (8.7%) | 1 (3.3%) | .573 |
| ICU readmission (n, %) | 4 (17.4%) | 3 (10.0%) | .451 |
| Rethoracotomy (n, %) | 4 (17.4%) | 3 (10.0%) | .451 |
| Cardiac tamponade (n, %) | 1 (4.3%) | 2 (6.7%) | 1.000 |
| **Length of stay** | | | |
| ICU length of stay (days) | 3.0 [2.0-4.5] | 2.5 [1.0-7.0] | .387 |
| Hospital length of stay (days) | 8.0 [5.0-12.0] | 8.0 [6.0-15.0] | .614 |
| *All values are presented as median [IQR], unless stated otherwise.*  **Abbreviations:** ECMO = extracorporeal membrane oxygenation; ICU = intensive care unit; IQR = interquartile range.  ^1^ Detailed definitions of all outcomes are provided in the Supplementary Material (S1). | | | |

**Supplementary Table S5.3.1: per protocol analyses for complete cohort (2020-2025), baseline characteristics**

| **Parameter** | Conventional (n=15) | Total body perfusion (n=30) | p-value |
| --- | --- | --- | --- |
| **Demographics** | | | |
| Male (n, %) | 11 (73.3%) | 19 (63.3%) | .737 |
| Age (years) | 63.3 [57.4,67.8] | 65.4 [61.9,73.7] | .142 |
| BMI (kg/m^2^) | 25.5 [23.0-27.4] | 25.6 [23.2-29.5] | .485 |
| EuroSCORE II | 5.6 [3.4-10.5] | 5.0 [3.5-7.0] | .684 |
| **Comorbidities and cardiac function** | | | |
| Diabetes (n, %) | 1 (6.7%) | 2 (6.7%) | 1.000 |
| Preoperative serum creatinine (umol/L) | 96.0 [77.5-105.0] | 87.5 [70.0,100.0] | 0.386 |
| Preoperative eGFR  (mean ± SD, ml/min) | 76.8 ± 24.7 | 78.1 ± 19.8 | .857 |
| LVEF (%) | 55 [53-55] | 55 [46.2-58.5] | .941 |
| **Operative characteristics** | | | |
| Reoperation (n, %) | 6 (40.0%) | 5 (16.7%) | .140 |
| Chronic aortic arch dissection (n, %) | 0 (0%) | 6 (20%) | .131 |
| Active infective endocarditis, prosthetic infection, or mycotic aneurysm at time of surgery (n, %) | 0 (0%) | 2 (6.7%) | .545 |
| *All values are presented as median [IQR], unless stated otherwise.*  Abbreviations: BMI = Body Mass Index; eGFR = estimated glomerular filtration rate (as estimated by CKD-EPI); IQR = interquartile range; LVEF = left ventricular ejection fraction; SD = standard deviation | | | |

**Supplementary Table S5.3.2: per protocol analyses for complete cohort (2020-2025), intraoperative characteristics**

| Parameter | Conventional (n=15) | Total body perfusion (n=30) | p-value |
| --- | --- | --- | --- |
| **Concomitant procedures** | | | |
| Concomitant aortic root procedure (n, %) | 5 (33.3%) | 13 (43.3%) | .189 |
| Any concomitant cardiac surgery (n, %) | 3 (20.0%) | 21 (70.0%) | **.043*** |
| Total arch replacement (n, %) | 11 (73.3%) | 27 (90.0%) | .199 |
| FET/ET (n, %) | 9 (60.0%) | 13 (43.3%) | .460 |
| **Operative parameters** | | | |
| Lowest temperature (°C) | 26.0 [25.1-27.2] | 29.4 [28.0-30.0] | **<.001*** |
| ECC duration  (mean ± SD minutes) | 258.9 ± 58.2 | 240.4 ± 49.3 | .305 |
| Myocardial ischemia time  (mean ± SD minutes) | 123.7 ± 52.1 | 60.0 ± 42.3 | **<.001*** |
| Circulatory arrest (n, %) | 13 (86.7%) | 3 (10.0%) | **<.001*** |
| Circulatory arrest time (minutes) | 46.0 [38.5-55.0] | 0.0 [0.0-9.0] | **<.001*** |
| *All values are presented as median [IQR], unless stated otherwise.*  Abbreviations: ECC = extracorporeal circulation; ET = elephant trunk; FET = frozen elephant trunk; IQR = interquartile range. | | | |

**Supplementary Table S5.3.3: per protocol analyses for complete cohort (2020-2025), biochemical outcomes**

| Parameter | Reference | Conventional (n=15) | Total body perfusion (n=30) | p-value |
| --- | --- | --- | --- | --- |
| **Peak lactate levels** | | | | |
| Intraoperatively (mmol/L) | 0.6-2.4 | 4.3 [3.0-4.8] | 2.2 [1.5-3.3 | **.008*** |
| 48h postoperatively (mmol/L) |  | 4.3 [3.0-6.1] | 2.8 [1.6-4.6] | .131 |
| 48h postoperatively (including intra-operative values) (mmol/L) |  | 3.8 [2.0-6.3] | 2.4 [1.4-3.9] | .054 |
| **Peak concentrations cardiac biomarkers 48h postoperatively** | | | | |
| CK (U/L) | <160 | 943.0 [670.0-2875.0] | 737.0 [466.2-1231.2] | .220 |
| CKMB (ug/L) | <2.9 | 45.5 [31.1-287.0] | 39.7 [30.4-80.2] | .354 |
| hsTnT (ng/L) | <14 | 5739.0 [5739.0-5739.0] | 2011.0 [929.0-2740.0] | .180 |
| **Renal biomarkers** | | | | |
| Peak serum creatinine concentration 48h postoperatively (umol/L) | 50-100 | 183.0 [85.5-224.5] | 105.0 [87.0-130.0] | .230 |
| Maximum creatinine elevation from baseline |  | 63.0 [5.0-118.5] | 17.5 [4.5-47.0] | .139 |
| *All values are presented as median [IQR], unless stated otherwise.*  Abbreviations: CK = creatinine kinase; CK-MB = creatinine kinase-M; hsTnT = high-sensitivity troponin T; IQR = interquartile range. | | | | |

**Supplementary Table S5.3.4: per protocol analyses for complete cohort (2020-2025), clinical outcomes**

| Parameter **^1^** | Conventional (n=15) | Total body perfusion (n=30) | p-value |
| --- | --- | --- | --- |
| **Mortality** | | | |
| In-hospital mortality (n, %) | 7 (46.7%) | 3 (10.0%) | **.009*** |
| **Cardiovascular complications** | | | |
| Myocardial infarction (n, %) | 1 (6.7%) | 1 (3.3%) | 1.000 |
| **Respiratory complications** | | | |
| Pulmonary infection (n, %) | 1 (6.7%) | 8 (26.7%) | .234 |
| Respiratory insufficiency (n, %) | 3 (20.0%) | 2 (6.7%) | .315 |
| **Neurological complications** | | | |
| Permanent neurological dysfunction (n, %) | 1 (6.7%) | 1 (3.3%) | 1.000 |
| Transient neurological dysfunction (n, %) | 1 (6.7%) | 2 (6.7%) | 1.000 |
| Total stroke incidence (n, %) | 2 (13.3%) | 3 (10.0%) | 1.000 |
| **Renal and gastrointestinal complications** | | | |
| Acute renal failure (n, %) | 7 (46.7%) | 3 (13.3%) | **.026*** |
| Gastrointestinal complications (n, %) | 3 (20.0%) | 1 (3.3%) | .101 |
| **ICU management and surgical complications** | | | |
| ECMO (n, %) | 1 (6.7%) | 1 (3.3%) | 1.000 |
| ICU readmission (n, %) | 3 (20.0%) | 3 (10.0%) | .384 |
| Rethoracotomy (n, %) | 3 (20.0%) | 3 (10.0%) | .384 |
| Cardiac tamponade (n, %) | 0 (0.0%) | 2 (6.75) | .545 |
| **Length of stay** | | | |
| ICU length of stay (days) | 4.0 [2.5-4.5] | 2.5 [1.0-7.0] | .210 |
| Hospital length of stay (days) | 8.0 [3.5-12.0] | 8.0 [6.0-15.0] | .507 |
| *All values are presented as median [IQR], unless stated otherwise.*  **Abbreviations:** ECMO = extracorporeal membrane oxygenation; ICU = intensive care unit; IQR = interquartile range.  ^1^ Detailed definitions of all outcomes are provided in the Supplementary Material (S1). | | | |

**Supplementary Figure S1: sensitivity analyses for complete cohort 2014-2025 (as-treated and per-protocol 1-year survival Kaplan-Meier curves)**

**
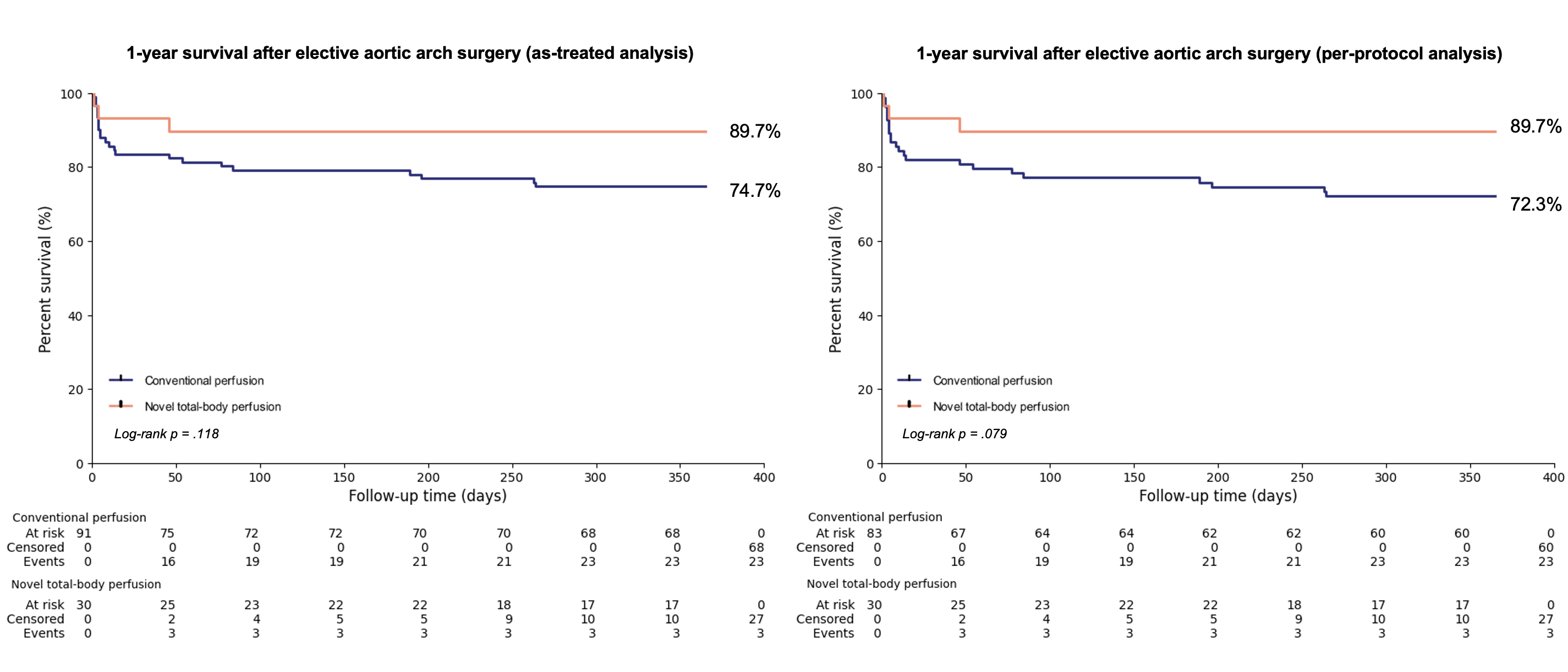
**

**Supplementary figure S1:** Kaplan–Meier curves for 1-year survival by perfusion strategy for complete cohort (2014-2025), shown as sensitivity analyses for the as-treated (left) and per-protocol (right) populations.

**Supplementary Figure S2: sensitivity analyses for 2020-2025 cohort (intention-to-treat, per-protocol, as-treated 1-year survival Kaplan Meier curves)**


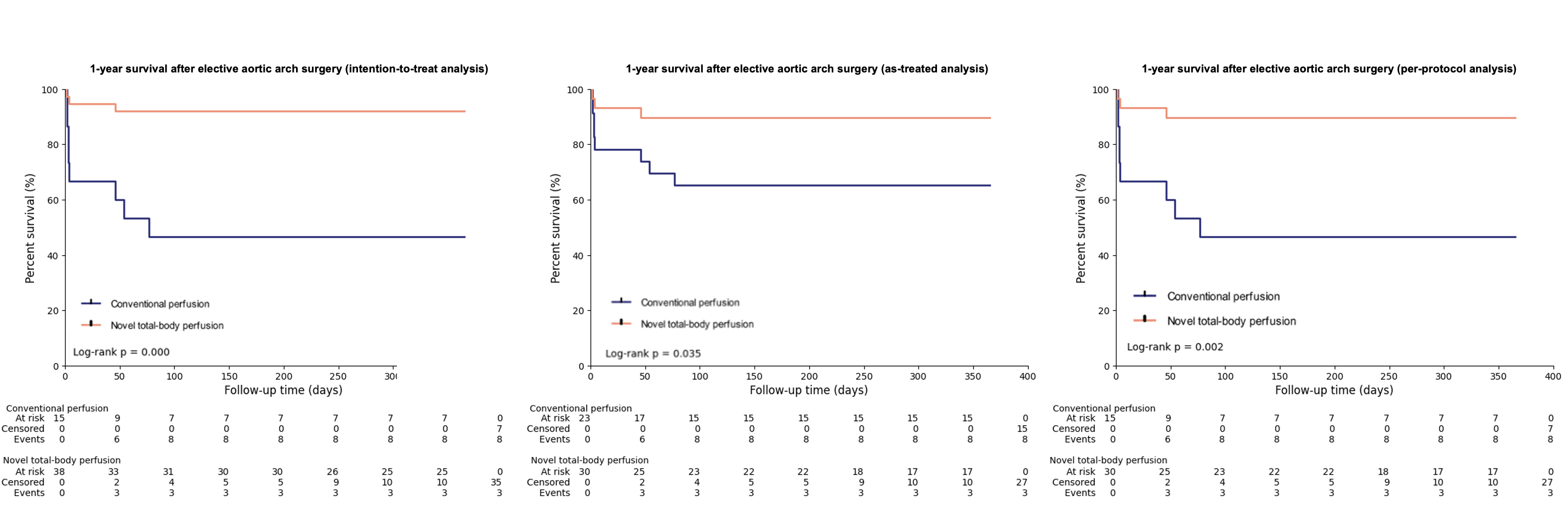


**Supplementary figure S2:** Kaplan–Meier curves for 1-year survival by perfusion strategy for contemporary cohort (2020-2025), shown as sensitivity analyses for the intention-to-treat (left), per-protocol (middle), and as-treated (right) populations.

**Supplementary Figure S3: Landmark-analysis**

**
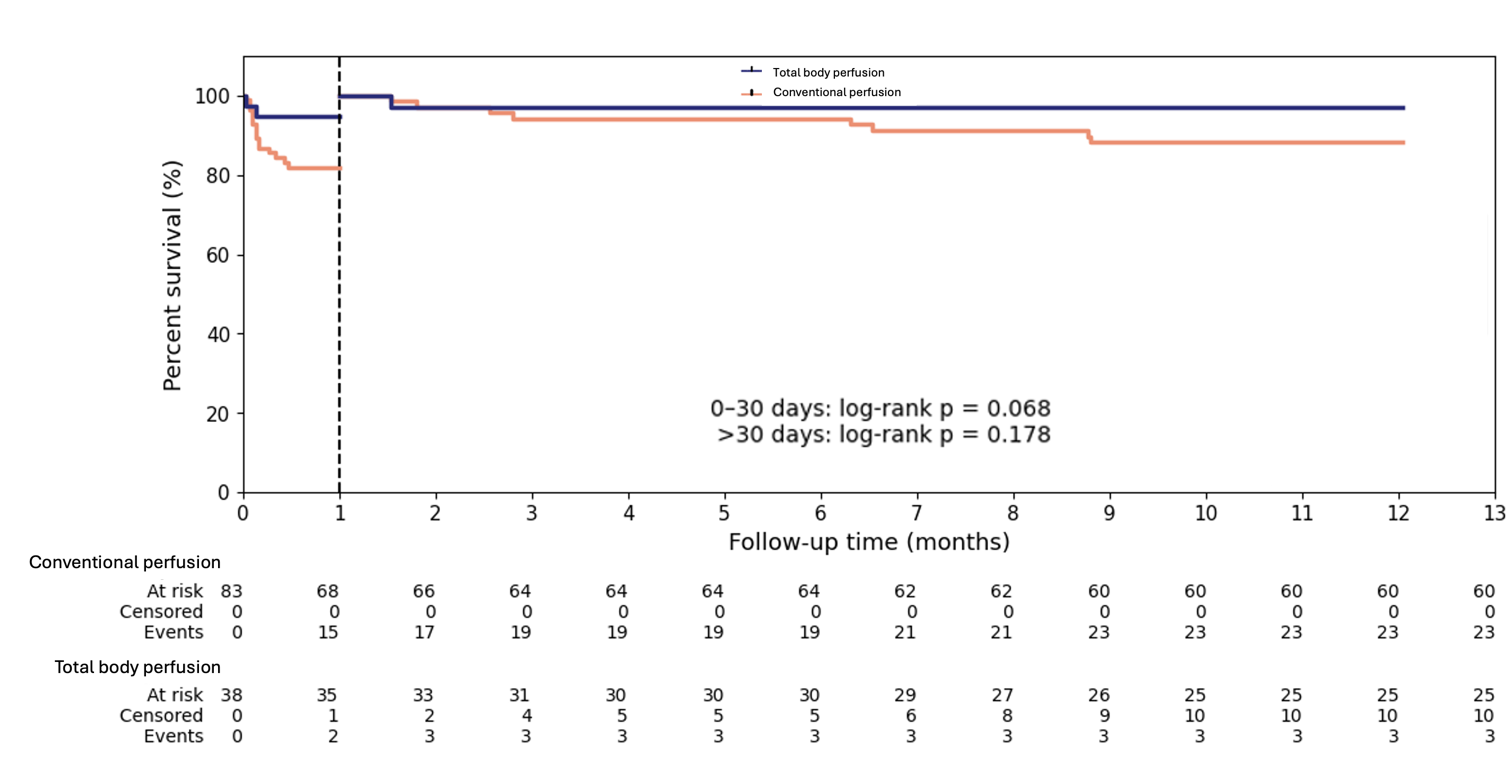
**

**Supplementary figure S3:** Kaplan–Meier landmark analysis for events before and after the 30-day time point.
